# Effects of closed loop ventilation on ventilator settings, patient outcomes and ICU staff workloads – a systematic review

**DOI:** 10.1097/EJA.0000000000001972

**Published:** 2024-03-04

**Authors:** Robin L. Goossen, Marcus J. Schultz, Edda Tschernko, Michelle S. Chew, Chiara Robba, Frederique Paulus, Pim L.J. van der Heiden, Laura A. Buiteman-Kruizinga

**Affiliations:** From the Department of Intensive Care, Amsterdam University Medical Centres, location ‘AMC’, Amsterdam, the Netherlands (RLG, MJS, FP, LAB-K), Mahidol–Oxford Tropical Medicine Research Unit (MORU), Mahidol University, Bangkok, Thailand (MJS), Nuffield Department of Medicine, University of Oxford, Oxford, UK (MJS), Department of Anaesthesia, General Intensive Care and Pain Management, Medical University Wien, Vienna, Austria (MJS, ET), Department of Anaesthesia and Intensive Care, Biomedical and Clinical Sciences, Linköping University, Linköping, Sweden (MSC), Unit of Anaesthesia and Intensive Care, IRCCS Policlinico San Martino, Genoa, Italy (CR), ACHIEVE, Centre of Applied Research, Amsterdam University of Applied Sciences, Faculty of Health, Amsterdam (FP), Department of Intensive Care, Reinier de Graaf Hospital, Delft, the Netherlands (PL.J.H, LAB-K)

## Abstract

**BACKGROUND:**

Lung protective ventilation is considered standard of care in the intensive care unit. However, modifying the ventilator settings can be challenging and is time consuming. Closed loop modes of ventilation are increasingly attractive for use in critically ill patients. With closed loop ventilation, settings that are typically managed by the ICU professionals are under control of the ventilator's algorithms.

**OBJECTIVES:**

To describe the effectiveness, safety, efficacy and workload with currently available closed loop ventilation modes.

**DESIGN:**

Systematic review of randomised clinical trials.

**DATA SOURCES:**

A comprehensive systematic search in PubMed, Embase and the Cochrane Central register of Controlled Trials search was performed in January 2023.

**ELIGIBILITY CRITERIA:**

Randomised clinical trials that compared closed loop ventilation with conventional ventilation modes and reported on effectiveness, safety, efficacy or workload.

**RESULTS:**

The search identified 51 studies that met the inclusion criteria. Closed loop ventilation, when compared with conventional ventilation, demonstrates enhanced management of crucial ventilator variables and parameters essential for lung protection across diverse patient cohorts. Adverse events were seldom reported. Several studies indicate potential improvements in patient outcomes with closed loop ventilation; however, it is worth noting that these studies might have been underpowered to conclusively demonstrate such benefits. Closed loop ventilation resulted in a reduction of various aspects associated with the workload of ICU professionals but there have been no studies that studied workload in sufficient detail.

**CONCLUSIONS:**

Closed loop ventilation modes are at least as effective in choosing correct ventilator settings as ventilation performed by ICU professionals and have the potential to reduce the workload related to ventilation. Nevertheless, there is a lack of sufficient research to comprehensively assess the overall impact of these modes on patient outcomes, and on the workload of ICU staff.

**KEY POINTS**

- Closed loop ventilation automates ventilator settings that are typically manually adjusted by the user during conventional ventilation.
- This systematic review identified 51 studies regarding six closed loop ventilation modes.
- Closed loop ventilation is at least as effective in choosing lung protective ventilator settings as ventilation performed by ICU professionals.
- Closed loop ventilation has the potential to decrease ICU staff workload, and even improve patient outcomes, although these findings are limited by underpowered study designs.

## Introduction

Mechanical ventilation is a key element of respiratory support in critically ill patients with respiratory failure. In the early years of critical care, the one–single goal of mechanical ventilation was to provide sufficient gas exchange, often targeting physiological levels of arterial partial pressures of oxygen (*p*aO_2_) and carbon dioxide (*p*aCO_2_).^1^ In the last decades, the goals of ventilation shifted towards lung protection, even if this jeopardised the initial ventilatory targets (e.g. by applying permissive hypercapnia, to reduce tidal volume and plateau pressure).^2^ While so-called lung protective ventilation has become the standard of care,^3^ its application in clinical practice can be challenging and time consuming; achieving the ventilatory targets requires complex titrations of ventilator settings according to the individual needs of patients, which change over time. There is clearly no ‘one–size–fits–all’, and constant individualisation and titration of ventilatory settings are required mandating the use of sometimes complex bedside calculations. Currently, lung protective ventilation includes a low tidal volume (*V*_T_), to prevent volutrauma and barotraumas; low pressures and energy, to avoid energy trauma; and restricted oxygen, to minimise chemotrauma.

Automated, or closed loop modes of ventilation, are increasingly attractive for use in the ICU.^4^ Ventilator settings that are typically manually adjusted by the user during conventional ventilation can, once the targets are manually set, be controlled by the software during closed loop ventilation. Closed loop ventilation has the potential to optimise ventilator settings, to increase safety of ventilation, and even to improve patient outcomes.^5,6^ Closed loop ventilation might also reduce ICU nursing and medical staff workload, through immediate reaction to patients’ changing demands.^7^ This is particularly interesting when faced with increasing challenges due to shortages in ICU nursing staff,^8^ and especially in extreme situations as seen in the recent coronavirus disease 2019 (COVID-19) pandemic when large numbers of patients required invasive mechanical ventilation.

We present the results of a systematic search of the literature for publications on randomised clinical trials of closed loop ventilation that focused on effectiveness in providing lung protective ventilation and settings, safety, patient outcomes related efficacy and ICU staff workloads (Table 1). We hypothesised that currently available closed loop ventilation modes are effective, well tolerated and efficacious, while reducing the ICU staff workloads.

**Table 1:** Definitions used for Outcome parameters

Outcome parameters	Definition
Effectiveness	The ability of the closed loop mode to institute appropriate settings as reflected by VT, ΔP, MP or FiO_2_, and to provide lung protective ventilation
Safety	Any adverse event, or discontinuation or change in a ventilator setting related to the closed loop mode under investigation because of unacceptable changes in clinical parameters
Efficacy	The effect of the closed loop mode on patient–related outcomes such as mortality, duration of ventilation, and ICU and hospital lengths of stay
Workload	The effect on staff workload such as the number of manual interventions to ventilator settings, or the number of alarms

## Materials and methods

### Search details

We conducted a literature search using various combinations of keywords and MeSH terms, including ‘Interactive Ventilatory Support’, ‘Respiration, Artificial’, ‘Automation’, ‘closed loop ventilation’, ‘automated ventilation’, ‘mechanical ventilation’ and ‘explicit computerized protocols’ in PubMed, Embase and the Cochrane Central register of Controlled Trials (CENTRAL). Inclusion criteria were randomised clinical trials that studied the effect of closed loop ventilation modes on ventilator settings, patient outcomes and ICU staff workload. We used no time or language restrictions, and included publications of studies in all patient categories, including paediatric and adult ICU cohorts. The reference lists of studies and systematic reviews identified by the search were used to find additional reports that may have been missed by the original search. The search was registered at PROSPERO with registration number CRD42023446174, and a final search was performed in January 2023.

Publications identified by the search were screened for eligibility by two independent investigators (RLG and LAB-K) by reading the titles and abstracts. If a study was considered potentially eligible, the full text was obtained, and reviewed for using the predefined inclusion and exclusion criteria.

### Selection of studies

A publication was eligible if reporting on a randomised clinical trial of closed loop ventilation; in invasively ventilated paediatric or adult ICU patients; and reporting on aspects regarding effectiveness, safety, efficacy or workload.

We selected studies that tested either SmartCare (Dräger, Lübeck, Germany), Adaptive Support Ventilation (ASV) or INTELLiVENT–ASV (Hamilton Medical, Bonaduz, Switzerland), Neurally Adjusted Ventilatory Assist (NAVA) (Getinge, Goteborg, Sweden), Proportional Assist Ventilation Plus (PAV+) (Puritan Bennett, Minneapolis, USA) and Avea–CLiO_2_ (CareFusion, Yorba Linda, California, USA). For details on these closed loop modes, see Fig. 2.

We excluded reports on studies of noninvasive ventilation, and ventilation in another setting than the ICU, that is ventilation in an emergency department or in an operating room.

### Extracted data

From each study, we collected the following data: patient characteristics, duration of ventilation or study intervention, and the investigated mode of closed loop and conventional ventilation. Data regarding effectiveness included ventilator settings and ventilation parameters such as tidal volume (*V*_T_), driving pressure (Δ*P*), mechanical power (MP) or fraction of inspired oxygen (FiO_2_). The rationale for choosing these effectiveness parameters in the light of closed loop ventilation and the current challenges in lung protective ventilation can be found in the Supplement. Data regarding safety included any adverse event, or discontinuation or change in a ventilator setting related to the closed loop mode under investigation because of unacceptable change in clinical parameters. Efficacy data included patient-related outcomes, such as duration of ventilation, length of stay in ICU or mortality rates. Data regarding workload included the number of manual interventions at the ventilator, or the number of alarms.

### Risk of bias and study quality

For each study, information was collected for the assessment of the risk of bias. The Cochrane Collaboration's tool for assessing risk of bias was used to assess the risk of bias for the included studies.^9^

We also calculated the fragility index for studies having a statistically significant dichotomous primary outcome,^10,11^ and compared them with the number of patients lost to follow-up for that endpoint in order to assess the robustness of the study results; the fragility index calculates the number of patients required to lose statistical significance.^12^

### Reporting

Data were reported as medians with interquartile ranges or means with standard deviations. For each study that reported a dichotomous primary endpoint, the fragility index and the number of patients lost to follow-up were reported. We did not perform a meta-analysis of the studies identified by the search, because the studies used various outcome measures, and had different study designs and durations.

## Results

### Search results and risk of bias

**Fig. 1:**
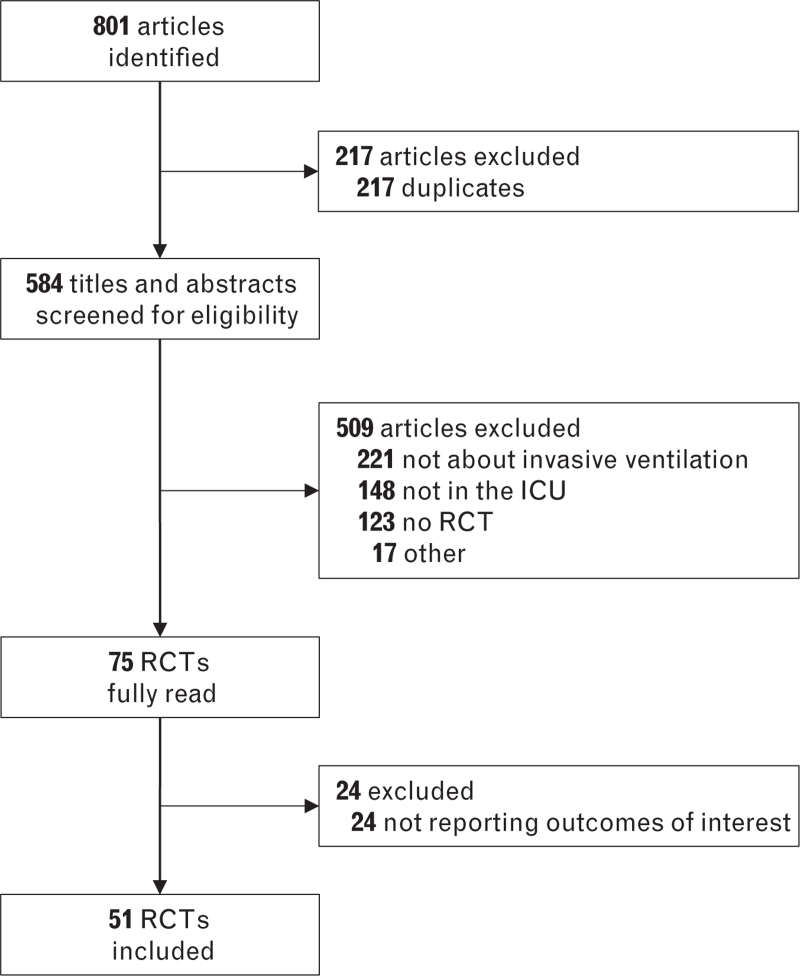
Search results.

The search identified a total of 801 studies; after removal of duplicates and screening for eligibility, 45 studies in adult^13–57^ and six studies in paediatric patients^58–63^ were considered for this analysis (Fig. 1 and eTable S1). Thirty-five studies had a parallel randomised design^13–19,21,23,25–31,33,34,36,38–40,43–57,62,63^; 12 had a crossover randomised design.^20,22,24,32,35,37,41,42,58–61^ Only five studies were multicentre investigations.^23,33,36,38,39^ Most studies were performed in critically ill patients with respiratory failure^15,16,18,22,24,26,27,29–31,33,35,36,39,41,46,47,54,62^; twelve studies in patients after cardiac surgery^13,17,25,28,34,48–51,57^ or general surgery,^20,23,43^ four in difficult to wean patients,^19,32,38,40,56^ four in COPD patients^14,21,44,45^ and four in ARDS patients.^37,42,53,55^ Nine studies tested SmartCare,^14,15,18,19,23,32,47,54,63^ fourteen studies ASV,^13,17,21,42,43,45,46,48–53,55^ eight studies INTELLiVENT–ASV,^22,24,25,27,28,30,34,35^ eight studies NAVA,^20,36–41,62^ six studies PAV+,^16,26,29,31,33,44^ three studies Avea–CLiO_2_^58–60^ and one closed loop FiO_2_ titrations during ASV.^61^ Observation duration differed substantially between studies (eTable S1).

As blinding of personnel was not possible due to the nature of the intervention, risk of performance bias was high in all studies (eFigure S1). In most studies, it was unclear how detection bias was avoided. Allocation concealment was used in 24 studies to reduce the risk of selection bias. The fragility index could be calculated in five studies and varied between 0 and 18 (eTable S2,).

**Fig. 2:**
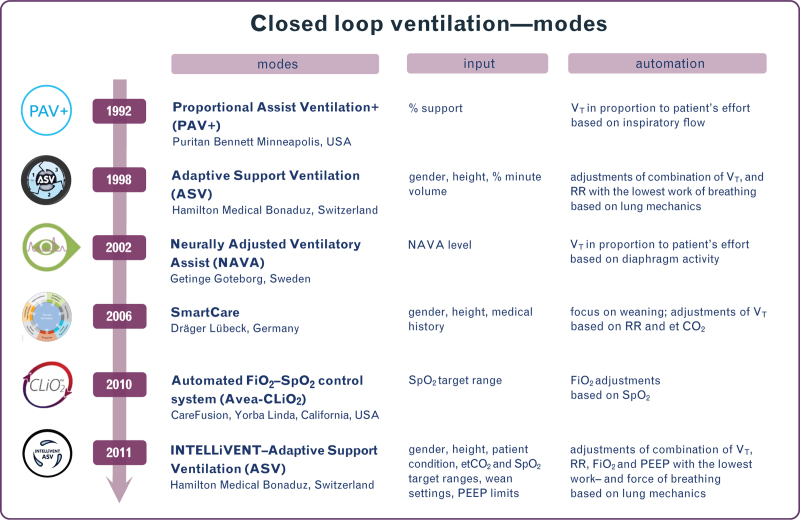
Closed loop ventilation modes.

etCO_2_, end-tidal carbon dioxide; FiO_2_, fraction of inspired oxygen; PEEP, positive end expiratory pressure; RR, respiratory rate; SpO_2_, pulse oximetry; *V*_T_, tidal volume.

### Effectiveness

**Fig. 3:**
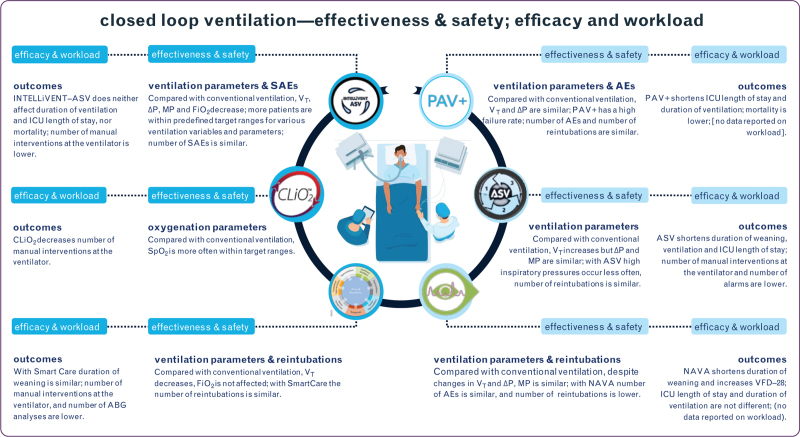
Summary of primary and secondary endpoints with significant results, reported for effectiveness, safety, efficacy and workload with closed loop ventilation. See text for details.

With SmartCare, *V*_T_ decreased in difficult to wean patients^32^ but was not affected in critically ill patients^47^ compared with conventional ventilation (Table 2, Fig. 3 and eTable S3). With ASV, *V*_T_ increased in cardiac surgery patients^17^ and in ARDS patients.^42^ ASV decreased *V*_T_ in ARDS patients,^55^ but did not affect *V*_T_ in cardiac surgery^48,51^ and COPD patients.^21,47^ INTELLiVENT-ASV led to a lower *V*_T_^22,25,34^ in cardiac surgery and unselected ICU patients, but *V*_T_ increased^24^ or was unaffected in general ICU patients.^27,30,35^ With NAVA, *V*_T_ decreased in ARDS patients.^37^ NAVA did not affect *V*_T_ in abdominal surgery patients.^20^ PAV+ did not affect *V*_T_ in a general ICU population.^16,29,31^

**Table 2:** Effectiveness, safety, efficacy and workload with closed loop ventilation

Author	Year	Ref	Patients	Closed loop mode tested	Effectiveness	Safety	Efficacy	Workload
Jiang *et al.*	2006	^14^	38 COPD patients	SmartCare	–	–	↑	↑
Stahl *et al.*	2009	^18^	60 patients needed ventilation >24 h	SmartCare	–	=	↑	=
Ma *et al.*	2010	^19^	62 difficult to wean patients	SmartCare	↑	–	↑	↑
Rose *et al.*	2008	^15^	102 patients needed ventilation >24 h	SmartCare	–	–	=	–
Schädler *et al.*	2012	^23^	300 surgical patients needed ventilation >9 h	SmartCare	–	–	=	–
Burns *et al.*	2013	^54^	92 critically ill patients	SmartCare	–	=	↑	–
Jouvet *et al.*	2013	^63^	30 unselected paediatric patients	SmartCare	–	=	↑	–
Liu *et al*	2013	^56^	39 difficult to wean patients	SmartCare	–	=	↑	–
Taniguchi *et al.*	2015	^47^	70 critically ill patients	SmartCare	=	=	↓	–
Grieco *et al.*	2018	^32^	30 difficult to wean patients	SmartCare	↑	–	–	–
Sulzer *et al.*	2001	^57^	36 cardiac surgery patients	Adaptive Support Ventilation	–	–	↑	–
Petter *et al.*	2003	^13^	30 cardiac surgery patients	Adaptive Support Ventilation	–	=	=	↑
Dongelmans *et al.*	2009	^17^	128 cardiac surgery patients	Adaptive Support Ventilation	=	–	=	–
Kirakli *et al.*	2011	^21^	97 COPD patients	Adaptive Support Ventilation	=	–	↑	–
Agarwal *et al.*	2013	^55^	48 ARDS patients	Adaptive Support Ventilation	↑	–	=	–
Celli *et al.*	2014	^43^	20 abdominal surgery patients	Adaptive Support Ventilation	–	–	↑	↑
Mohamed *et al.*	2014	^45^	50 COPD patients	Adaptive Support Ventilation	–	=	↑	–
Kirakli *et al.*	2015	^46^	229 critically ill patients	Adaptive Support Ventilation	–	–	=	↑
Zhu *et al.*	2015	^48^	53 cardiac surgery patients	Adaptive Support Ventilation	=	=	↑	–
Yazdannik *et al.*	2016	^49^	64 cardiac surgery patients	Adaptive Support Ventilation	–	–	↑	–
Moradian *et al.*	2017	^50^	115 cardiac surgery patients	Adaptive Support Ventilation	–	↑	↑	–
Eremenko *et al.*	2020	^51^	78 cardiac surgery patients	Adaptive Support Ventilation	=	=	=	↑
Baedorf Kassis *et al.*	2022	^42^	20 ARDS patients	Adaptive Support Ventilation	=	=	–	–
Sehgal *et al.*	2022	^52^	48 envenomation patients	Adaptive Support Ventilation	–	–	=	–
Soydan *et al.*	2022	^61^	30 critically ill paediatric patients	Adaptive Support Ventilation with closed–loop FiO_2_ titration	↑	↑	–	↑
Zhang *et al.*	2022	^53^	100 ARDS patients	Adaptive Support Ventilation	–	–	↑	–
Arnal *et al.*	2012	^22^	50 critically ill patients	INTELLiVENT–ASV	↑	=	=	–
Clavieras *et al.*	2013	^24^	14 critically ill patients	INTELLiVENT–ASV	=	–	=	–
Lellouche *et al.*	2013	^25^	60 cardiac surgery patients	INTELLiVENT–ASV	↑	–	=	↑
Bialais *et al.*	2016	^27^	80 critically ill patients	INTELLiVENT–ASV	↑	–	=	↑
Fot *et al.*	2017	^28^	40 cardiac surgery patients	INTELLiVENT–ASV	=	–	=	↑
Arnal *et al.*	2018	^30^	60 critically ill patients	INTELLiVENT–ASV	=	–	=	↑
De Bie *et al.*	2020	^34^	220 cardiac surgery patients	INTELLiVENT–ASV	↑	↑	=	–
Chelly *et al.*	2022	^35^	265 critically ill patients	INTELLiVENT–ASV	=	↑	=	↑
Coisel *et al.*	2010	^20^	15 abdominal surgery patients	Neurally–adjusted Ventilatory Assist	↑	↓	–	–
Demoule *et al.*	2016	^36^	128 patients with ARF	Neurally–adjusted Ventilatory Assist	–	–	=	–
Diniz–Silva *et al.*	2020	^37^	20 ARDS patients	Neurally–adjusted Ventilatory Assist	=	–	–	–
Hadfield *et al.*	2020	^38^	72 difficult to wean patients	Neurally–adjusted Ventilatory Assist	–	–	↑	–
Liu *et al.*	2020	^40^	47 difficult to wean patients	Neurally–adjusted Ventilatory Assist	–	–	↑	–
Kacmarek *et al.*	2020	^39^	306 patients with ARF	Neurally–adjusted Ventilatory Assist	–	↑	↑	–
Cammarota *et al.*	2022	^41^	16 patients with AHRF	Neurally–adjusted Ventilatory Assist	=	–	–	–
Xirouchaki *et al.*	2008	^16^	208 critically ill patients	Proportional Assist Ventilation+	=	=	–	–
Elganady *et al.*	2014	^44^	60 COPD patients	Proportional Assist Ventilation+	–	–	↑	–
Kallio *et al.*	2015	^62^	170 critically ill paediatric patients	Proportional Assist Ventilation+	–	=	=	–
Teixeira *et al.*	2015	^26^	160 patients needed controlled ventilation >24 h	Proportional Assist Ventilation+	–	–	=	–
Bosma *et al.*	2016	^29^	50 patients needed ventilation >36 h	Proportional Assist Ventilation+	=	=	=	–
Botha *et al.*	2018	^31^	50 patients needed controlled ventilation >24 h	Proportional Assist Ventilation+	↑	=	↑	–
Delgado *et al.*	2019	^33^	102 patients with ARF	Proportional Assist Ventilation+	–	↓	=	–
Claure *et al.*	2011	^58^	32 preterm infants	Avea–CLiO2	↑	↑	–	↑
Lal *et al.*	2015	^59^	27 preterm infants	Avea–CLiO2	↑	↑	–	↑
Kaam *et al.*	2015	^60^	80 preterm infants	Avea–CLiO2	↑	↑	–	↑

AHRF, acute hypoxemic respiratory failure; ARDS, acute respiratory distress syndrome; ARF, acute respiratory failure; ASV, adaptive support ventilation; COPD, chronic obstructive pulmonary disease; FiO_2_, fraction of inspired oxygen. ↑ improved, compared with conventional ventilation; = no difference, compared with conventional ventilation; ↓ worse, compared with conventional ventilation; – not reported

Clockwise the icons represent the following modes: PAV+, proportional assist ventilation; ASV, adaptive support ventilation; NAVA, neurally adjusted ventilatory assist; SmartCare; Avea–CLiO_2_; INTELLiVENT–ASV, INTELLiVENT adaptive support ventilation. Abbreviations: DP, driving pressure; (S)AE, (serious) adverse event; ABG, arterial blood gas analysis; MP, mechanical power; SpO_2_, peripheral oxygen saturation; VFD, ventilator-free days; *V*_T_, tidal volume.

ASV did not affect Δ*P* and MP in ARDS patients,^42^ but with INTELLiVENT–ASV, Δ*P* and MP were lower in cardiac surgery patients,.^34^ With NAVA, Δ*P* increased but MP decreased in hypoxemic respiratory failure patients.^41^ PAV+ did not affect Δ*P* in general ICU patients.^29^

Smartcare, ASV and NAVA did not affect FiO_2_ in critically ill patients,^47^ cardiac surgery patients^51^ and paediatric patients,^62^ respectively. INTELLiVENT–ASV reduced FiO_2_ in cardiac surgery patients^25,34^ and in critically ill patients,^22,24,27,30^ Avea–CLiO_2_ and a closed loop FiO_2_ controller available for use with ASV also increased time spent in preferred SpO_2_ ranges in paediatric patients.^58–61^

### Safety

SmartCare, ASV and INTELLiVENT–ASV resulted in a similar number of reported ‘adverse events’^18,35,42,45,47,48,51^ (Table 2, Fig. 3 and eTable S4). ASV was associated with lower peak pressures^47^ and a lower incidence of atelectasis.^50^ INTELLiVENT–ASV was associated with less hypoxemic events,^34,35^ and increased time spent in preferred SpO_2_ ranges in medical ICU patients.^35^ NAVA was associated with less extubation failure.^39^ PAV+ did not affect reintubation rates^31^ but had to be discontinued in a large proportion of patients.^33^ Avea–CLiO_2_ as well as a closed loop FiO_2_ controller available for use with ASV were associated with less hypoxemic events.^58–61^

### Efficacy

SmartCare was associated with shorter^54,56,63^ or similar weaning duration in various patient categories^14,15,18,23^ (Table 2, Fig. 3 and eTable S5). ASV^21,43,45,46,48,49,57^ and NAVA^40^ were also associated with shorter duration of weaning and shorter duration of ventilation, and NAVA with more ventilator free days.^38,39^ PAV+ was associated with shorter duration of ventilation, ICU and hospital length of stay^29,44^ and improved survival.^31^

### Workload

SmartCare,^18,19^ ASV,^43,46,51^ INTELLIVENT–ASV^25,27,28,30^ and Avea–CLiO_2_ were associated with fewer manual interventions at the ventilator^58–61^ (Table 2, Fig. 3 and eTable S6). SmartCare was associated with a lower number of blood gas analyses.^14^ ASV was associated with fewer alarms^13^ and less time spent at or approaching the ventilator.^51^

## Discussion

The findings of this systematic review can be summarised as follows: there are currently six commercially available closed loop ventilation modes for use in critically ill patients; their effectiveness (in terms of ventilator settings) and efficacy (in terms of patient outcomes), have been studied in various cohorts of patients; and safety (in terms of adverse events) has seldom been reported. In addition, the effect of these closed loop modes on workload of ICU staff has not yet been sufficiently researched.

Our analysis has several strengths. We conducted a comprehensive and unrestricted search. By reviewing the reference lists of the identified articles, we searched for additional studies that may not have been identified by the search. We applied clear inclusion and exclusion criteria for the selection of articles of interest. We checked the robustness of the study findings by comparing the fragility index with the number of patients lost to follow-up for binary clinical endpoints.

To our knowledge, this systematic review is the first to focus on all commercially available closed loop ventilation modes for use in the ICU, addressing several aspects of care and outcomes related to ventilation. The findings extend those of a previous review that focused only on INTELLiVENT–ASV.^5^ Although effectiveness is important in our assessment, we believe that from the four endpoints investigated, efficacy and safety, and in particular ICU staff workload, should always be considered when evaluating the advantages and disadvantages of any mode of closed loop ventilation.

Each closed loop ventilation mode seemed to be effective with regard to one or more aspects of lung protective ventilation, and some were even associated with a higher efficacy. Unfortunately, however, each study used different effectiveness endpoints, therefore an interaction with other ventilation parameters in potentially nonlung protective ranges could not be determined. Moreover, this hampered meta-analysis of the studies. Some of the included articles also reported opposite results related to ventilation parameters. This is possibly due to the different patient groups included in the study, to the study design or to the local use of the reported ventilation modes. Software changes did not occur for the different ventilation modes in a way that algorithms changed ventilation strategies leading to opposite ventilation variables. It is important to mention that most, if not all, studies were performed in centres with experience in invasive ventilation, meaning that standard ventilation care was most likely at a high level. Even while this may reduce the chance of showing superiority of the tested closed loop mode, with regard to effectiveness, most studies found the closed loop mode to be superior or at least as effective. On the contrary, with regard to efficacy endpoints, only some studies reported superiority. Herein, it should also be realised that most studies were small, and probably too small to have sufficient power to demonstrate superiority with respect to clinical outcomes.

Safety endpoints varied from adverse events that were predefined as a clinical endpoint, such as reintubation, to proportions of time spend outside of ‘safe’ zones of ventilation. In addition to effectiveness and efficacy, each study used other clinical safety endpoints. Severe adverse events, or adverse events, were never reported. Very probably, these were either not collected systematically, or simply not reported. The high failure rate of PAV+ (discontinuation of the automated mode) in one study was attributed to excessive sedation, high respiratory rate and high respiratory effort.^33^ Whereas this is an unfavourable event, it did not hamper patient safety. Future studies are needed to determine safety of closed loop ventilation, particularly in centres with less experience of invasive ventilation, and outside a research setting. In order to clinically interpret safety endpoints, details such as the reason for discontinuation, should be given.

Staff workload is difficult to capture, and thus far, there have been no studies of ICU staff workload related to ventilation. Our search identified only a small number of studies that reported on manual interventions, alarms, and the need for blood gas analysis. While these studies all showed a reduction of these three aspects, it remains uncertain if this truly reflects a reduction in ICU staff workload, during different phases of mechanical ventilation: for example, the weaning phase in particular, is seen as a labour-intensive phase of mechanical ventilation.^64^ We need better studies in the future that, for instance, capture nursing activities scores with metrics that encompass the majority of tasks of an ICU nurse, including those related to invasive ventilation.^65,66^

Closed loop ventilation can facilitate rapid and precise adjustments to ventilator settings. In ICU subpopulations, such as traumatic brain injury patients, strict and precise titration of *p*aCO_2_ and *p*aO_2_ values are fundamental in order to optimise intracerebral physiology.^67–69^ In practice, it is difficult and time consuming for the ICU staff to achieve this. Closed loop ventilation could help to achieve strict and precise titration, while considerably reducing workload.

This systematic review has limitations. In coherence with the articles included, this review displays a large variety in the endpoints, effectiveness and efficacy, hampering meta–analysis and with that, limiting conclusions on effectiveness, safety, efficacy and workload. In particular, safety reporting was scarce, albeit we expected this to be one of the most important endpoints to report in studying available closed loop ventilation modes. We did not reach out to the researchers in order to collect individual patient data on seldom reported endpoints such as safety and workload. Moreover, the fragility index as a measure of robustness of study results was only applicable for the minority of included studies and, where assessed, high fragility was found.

## Conclusion

The current commercially available closed loop ventilation modes are at least as effective compared with conventional ventilation. Safety is rarely reported, and efficacy has mostly been shown in small studies. The effect of closed loop ventilation on workload of ICU staff has not yet been sufficiently researched.

## Supplementary Material

Supplemental Digital Content
