# Supplementary material for: Effects of closed loop ventilation on ventilator settings, patient outcomes and ICU staff workloads – a systematic review
Source: Eur J Anaesthesiol. 2024 Mar 4;41(6):438–46. doi: 10.1097/EJA.0000000000001972 (PMC11064903; doi:10.1097/EJA.0000000000001972)
Supplement: Supplemental Digital Content [file ejanet-41-438-s001.docx]

Supplement to:

**Effects of Closed loop Ventilation on Ventilator Settings, Patient Outcomes and Nursing Staff Workloads – a systematic review**

Robin L. Goossen^1^, Marcus J. Schultz^1,2,3,4^, Edda Tschernko^4^, Michelle Chew^5,6^, Chiara Robba^7^, Frederique Paulus^1,8^, Pim L.J. van der Heiden^9^, Laura A. Buiteman–Kruizinga^1,9^

**Table of contents**

The ‘burden’ of lung protective ventilation 3

eTable S1 5

eTable S2 11

eTable S3 12

eTable S4 14

eTable S5 16

eTable S6 21

eFigure S1 23

Search details 24

References 25

**The ‘burden’ of lung protective ventilation**

Lung protective ventilation comes with challenges, as there is clearly no ‘one–size–fits–all’, and requires constant individualization and titration of ventilatory settings. Intensive monitoring of ventilation and oxygenation parameters in response to changes in patients’ individual needs is constantly needed. It mandates the use of sometimes complex bedside calculations. It also involves near constant adjustments and refinements to comply with the ever changing pulmonary conditions. This all makes it a difficult and time consuming strategy, i.e., it increases ICU staff workloads.

*Tidal volume*

The seminal randomized clinical trial named ARMA demonstrated that ventilation with a too high V_T_ is harmful especially when an exceptionally high V_T_ of ≥12 ml kg^-1^ predicted body weight (PBW), is being used in patients with acute respiratory distress syndrome (ARDS) [1]. After publication of this study, low V_T_ ventilation (LTVV) became standard of care. However, LTVV does not necessarily mean that V_T_ should always be < 6 ml kg^-1^ (PBW). First, in ARMA slightly higher V_T_ of up to 8 ml kg^-1^ PBW were allowed in patients with severe dyspnoea. Second, in patients with a relatively normal respiratory system compliance (C_RS_) using a higher V_T_, together with an increase in the respiratory rate (RR) to compensate for the decline in alveolar minute volume with low V_T_, may reduce mortality [2]. While it is probably best practice to use V_T_ close to 6 ml kg^-1^ PBW in patients with ARDS, and certainly in those patients without spontaneous breathing activity receiving controlled ventilation [3], it is much less certain what V_T_ to use in patients with spontaneous breathing activity during support ventilation, and in patients without ARDS [4].

*Driving pressure*

Several investigations suggest that a higher driving pressure (ΔP), the difference between plateau pressure (Pplat) and positive end expiratory pressure (PEEP) and representing the ratio of V_T_ to C_RS_, is associated with increased mortality [5, 6]. With the increased use of LTVV, ΔP probably already led to a decrease in ΔP over the years, but further reduction in ΔP could be achieved by adjusting PEEP. Use of higher PEEP may recruit collapsed alveoli. This increases the end expiratory lung volume and consequently ΔP will decrease. However, this may only happen in patients with recruitable lung lesions. In patients with lung lesions that are not, or less well recruitable, higher PEEP may result more in overdistension of open lung units than recruitment of collapsed alveoli [7, 8], actually increasing ΔP, and thereby potentially worsening outcomes.

*Mechanical power of ventilation*

Recent studies also suggest that the mechanical power of ventilation (MP), a summary parameter that includes V_T_, RR, maximum airway pressure and ΔP [9], reflecting the energy transferred from the ventilator to the respiratory system [10-12], is associated with mortality [13, 14]. Even short periods of high MP have associations with worse outcomes [15]. Targeting a low MP involves the use of complex bedside calculations and also decisions regarding which ventilation setting to prioritize in an attempt to reduce MP. Adjusting a setting that aimed at decreasing MP may require a change in another setting that paradoxically increases MP––for example, a decrease in V_T_ in an attempt to lower MP may require an increase in RR, but the higher RR will increase MP. Thus, targeting a low MP is complex.

*Oxygen*

Hyperoxia is increasingly recognized as harmful [16]. Several studies in critically ill patients showed a benefit of restricted oxygen use or lower oxygenation targets [17, 18]. Although not confirmed by other studies [19], it is now common practice to target lower oxygenation levels. A ventilation strategy aimed to lower oxygenation levels naturally comes with an increased risk of hypoxemia. Whether hyperoxia or hypoxia is more harmful is uncertain[20, 21]. In the specific population of acute brain injured patients, titration of PaO_2_ is even more fundamental; the brain is particularly susceptible to both hypoxia and hyperoxia, demonstrating a U-shaped curve related to mortality [22]. To avoid both hyperoxia and hypoxemia, frequent titrations of the fractions of inspired oxygen (FiO_2_) are needed. This is difficult and time consuming, especially in patients with rapid changes in oxygen demand.

|  | | | | | | | | |
| --- | --- | --- | --- | --- | --- | --- | --- | --- |
| **eTable S1.** Study characteristics | | | | | | | | |
| **first author (year)** | **ref** | **design** | ***n*** | **patient types** | **duration** | **closed loop**  **mode** | **conventional**  **mode** | **endpoints** |
| Sulzer *et al.* (2001) | [23] | parallel | 36 | cardiac  surgery | 193 [149–273] min | ASV | SIMV | duration of ventilation (E–cy); |
| Petter *et al.* (2003) | [24] | parallel | 30 | cardiac  surgery | 1.4 hours | ASV | SIMV | duration of ventilation (E–cy); number of high inspiratory pressure episodes (S); manual adjustments and alarms (W) |
| Jiang *et al.* (2006) | [25] | parallel | 38 | COPD | NR | SmartCare | PSV | duration of ventilation and weaning success (E–cy); number of ABG analyses (W) |
| Rose *et al.* (2008) | [26] | parallel | 102 | ventilation  >24 hours | 43 [6–169] | SmartCare | PSV | duration of ventilation and ICU and hospital LOS (E–cy) |
| Xirouchaki *et al*. (2008) | [27] | parallel | 208 | critically ill | 36 hours | PAV+ | PSV | V_T_ (E–ness); failure (S) |
| Dongelmans *et al*. (2009) | [28] | parallel | 128 | cardiac  surgery | 16.4 [12.5–20.8] hours | ASV | PCV  or PSV | V_T_ (E–ness); duration of ventilation and weaning success (E–cy) |
| Stahl *et al.* (2009) | [29] | parallel | 60 | ventilation  >24 hours | 0.64 [0.28–5.80] days | SmartCare | PSV | duration of weaning, duration of ventilation, ICU LOS and reintubation rate (E–cy); manual adjustments (W) |
| Ma *et al.* 2010) ^+^ | [30] | parallel | 62 | difficult to wean | N.R. | SmartCare | SIMV or  PSV | duration of weaning, ICU LOS (E–cy); SAEs (S); manual adjustments (W) |
| Coisel *et al.* 2010) | [31] | crossover | 15 | abdominal  surgery | 2x 24 hours | NAVA | PSV | V_T_ (E–ness); percentage of time spent in inadequate ventilation zones (S) |
| Kirakli *et al.* 2011) | [32] | parallel | 97 | COPD | 120 [72–264] hours | ASV | PSV | V_T_ (E–ness); duration of weaning, duration of ventilation and ICU LOS (E–cy) |
| Arnal *et al*.  (2012) | [33] | crossover | 50 | critically ill | 2 x 2 hours | INTELLiVENT–ASV | ASV | V_T_, FiO_2_, SaO_2_ (E–ness); failure (S) |
| Schädler *et al*. (2012) | [34] | parallel | 300 | post–surgical  ventilation  >9 hours | 24 [18–57] hours | SmartCare | PSV | duration of weaning, duration of ventilation, ICU LOS and mortality (E–cy) |
| Agarwal *et al.* (2013) | [35] | parallel | 48 | ARDS | 5 [3–11] days | ASV | VCV | V_T_ (E–ness); duration of ventilation, ICU and hospital LOS, mortality (E-cy) |
| Burns *et al.* (2013) | [36] | parallel | 92 | critically ill | 4.0 [2.0–7.0] days | SmartCare | PSV | reintubations (S); duration of weaning, duration of ventilation, ICU and hospital LOS, mortality (E-cy) |
| Clavieras *et al*. (2013) | [37] | crossover | 14 | critically ill | 2 x 24 hours | INTELLiVENT–ASV | PSV | V_T_, FiO_2_, SaO_2_ (E–ness); premature interruptions (S) |
| Lellouche *et al.* (2013) | [38] | parallel | 60 | cardiac  surgery | 4 hours | INTELLiVENT–ASV | VCV or PSV | V_T_, FiO_2_, (E–ness); premature interruptions, time within unsafe ventilation ranges (S); duration of weaning, ICU mortality (E–cy); manual adjustments (W) |
| Liu *et al.* (2013) | [39] | parallel | 39 | Difficult to wean | 138 [82.0–166.5] | SmartCare | PSV | reintubation (S); duration of weaning, duration of ventilation, ICU LOS and mortality (E–cy); |
| Celli *et al.* (2014) | [40] | parallel | 20 | abdominal surgery | 90 ± 13 min | ASV | P–SIMV | high respiratory pressure episodes (S); duration of weaning (E–cy); manual adjustments (W) |
| Elganady *et al.* (2014) | [41] | parallel | 60 | COPD | 6.33 ± 0.58 days | PAV+ | PSV | duration of ventilation, weaning success, ICU and hospital LOS (E–cy) |
| Mohamed *et al.* (2014) | [42] | parallel | 50 | COPD | 27.3 ± 12.3 hours | ASV | PSV | reintubation (S); duration of ventilation and weaning, weaning failure, ICU LOS 28–day mortality (E–cy) |
| Kirakli *et al.* (2015) | [43] | parallel | 229 | critically ill | 67 [43–94] hours | ASV | PSV or PCV | duration of ventilation and weaning, weaning success, 28–day mortality (E–cy); manual interventions (W) |
| Taniguchi *et al.* (2015) | [44] | parallel | 70 | critically ill | 3.5 (2.0–7.3) days | SmartCare | PSV | V_T_, FiO_2_ (E–ness); reintubation, ventilator related malfunction(S); duration of ventilation and weaning, weaning success (E–cy) |
| Teixeira *et al. (*2015) | [45] | parallel | 160 | ventilation  >24 hours | 30–90 min | PAV+ | PSV or  T–tube | premature interruptions (S); duration of ventilation, ICU LOS and mortality (E–cy) |
| Zhu *et al.* (2015) | [46] | parallel | 53 | cardiac surgery | 205 [141– 295] min | ASV | SIMV | V_T_, FiO_2_ (E–ness); reintubation (S); duration of ventilation, ICU and hospital LOS (E–cy) |
| Bialais *et al*. (2016) | [47] | parallel | 80 | critically ill | 48 hours | INTELLiVENT–ASV | N.S. | V_T_, FiO_2_ (E–ness); premature interruptions, time within unsafe ventilation ranges (S); duration of ventilation, ICU LOS and mortality (E–cy); manual and total adjustments (W) |
| Bosma *et al*. (2016) | [48] | parallel | 50 | ventilation  >36 hours | 4.9 [2.9–26.3] days | PAV+ | PSV | V_T_, ΔP (E–ness); AE (S); weaning duration, ICU and hospital LOS (E–cy) |
| Demoule *et al*. (2016) | [49] | parallel | 128 | ARF | 10.0 [7.0–16.0] days | NAVA | PSV | premature interruptions (S); VFD and mortality at day 28 (E–cy) |
| Yazdannik *et al.* (2016) | [50] | parallel | 64 | cardiac  surgery | 4.83 hours | ASV | SIMV | duration of ventilation, hospital LOS (E–cy) |
| Fot *et al. (*2017) | [51] | parallel | 40 | cardiac  surgery | 193 [115–309] min | INTELLiVENT–ASV | SIMV or  PSV | V_T_ (E–ness); premature interruptions, time within unsafe ventilation ranges (S); manual adjustments (W) |
| Moradian *et al.* (2017) | [52] | parallel | 115 | cardiac surgery | 296 ± 169 | ASV | SIMV | Incidence of atelectasis (S); duration of ventilation, ICU and hospital LOS (E–cy); manual adjustments and number of ABG analyses (W) |
| Arnal *et al.* (2018) | [53] | parallel | 60 | critically ill | 2.0 [1.0–4.0] days | INTELLiVENT–ASV | N.S. | V_T_, FiO_2_, (E–ness); duration of ventilation, duration of weaning, ICU LOS and mortality, 28 days mortality (E–cy); manual and total adjustments (W) |
| Botha *et al.* (2018) | [54] | parallel | 50 | ventilation  >24 hours | 9.3 days | PAV+ | PSV | VT, (E–ness); rescue ventilation, reintubation (S); time to successful weaning, ICU and hospital LOS and mortality (E–cy) |
| Grieco *et al*.  (2018) | [55] | crossover | 30 | difficult to wean | 2 x 3 hours | SmartCare | PSV | V_T_ (E–ness) |
| Delgado *et al*. (2018) | [56] | parallel | 102 | ARF | 3 days | PAV+ | VCV | failure rate (S); duration of ventilation, ICU and hospital LOS and mortality (E–cy) |
| de Bie *et al*.  (2020) | [57] | parallel | 220 | cardiac  surgery | 0.24 ± 0.17 days | INTELLiVENT–ASV | VCV or  PSV | V_T_, ΔP, MP, FiO_2_ (E–ness); premature interruptions, time within unsafe ventilation ranges (S); duration of ventilation, duration of weaning, ICU LOS and mortality, hospital mortality (E–cy) |
| Chelly *et al*. (2020) | [58] | parallel | 265 | critically ill | 2 x 30 min | INTELLiVENT–ASV | VCV,  BIPAP or PSV | V_T_, (E–ness); SAE, premature interruptions, time within unsafe ventilation ranges (S); manual adjustments (W) |
| Diniz–Silva *et al*. (2020) | [59] | crossover | 20 | ARDS | 2 x 3 hours | NAVA | PSV | V_T_ (E–ness) |
| Eremenko *et al*. (2020) | [60] | parallel | 78 | cardiac surgery | 267 ±76 min | ASV | SIMV | V_T_ (E–ness); reintubation (S); duration of ventilation, ICU and hospital LOS (E–cy); manual adjustments, approaches to the ventilator, time spent at the ventilator (W) |
| Hadfield *et al*. (2020) | [61] | parallel | 72 | difficult to wean | 4.9 [2.8–15.7] days | NAVA | PSV | VFD and mortality at day 28, ICU and hospital stay (E–cy) |
| Kacmarek *et al*. (2020) | [62] | parallel | 306 | ARF | 7.8 ± 8 hours | NAVA | VCV, PCV or PSV | weaning duration, VFD, mortality (E–cy) |
| Liu *et al*. (2020) | [63] | parallel | 47 | difficult to wean | 7.1 [5.0–12.5] hours | NAVA | PSV | adverse events (S); weaning duration, VFD, mortality (E–cy) |
| Cammarota *et al*. (2022) | [64] | crossover | 16 | AHRF | 2x 3 hours | NAVA | PSV | V_T_, ΔP, MP (E–ness) |
| Baedorf Kassis *et al*.  (2022) | [65] | crossover | 20 | ARDS | 2 x 1 to 2 hours | ASV | APV | V_T_, ΔP (E–ness); premature interruptions, adverse events (S) |
| Sehgal *et al.* (2022) | [66] | parallel | 48 | envenomation | 90.4 hours | ASV | VCV or PSV | post–extubation respiratory failure, ventilator–associated pneumonia, barotrauma (S); duration of ventilation, ICU and hospital LOS, mortality (E–cy) |
| Zhang *et al.* (2022) | [67] | parallel | 100 | ARDS | 258 ± 25 hours | ASV | SIMV | duration of ventilation, ICU LOS (E–cy) |
| Claure *et al.* (2011) | [68] | crossover | 32 | preterm | 2 x 24 hours | Avea–CliO_2_ | manual FiO_2_ control | time within unsafe SpO_2_ ranges, SAE (S); manual adjustments (W) |
| Jouvet *et al.* (2013) | [69] | parallel | 30 | unselected paediatric | 21 [3–142] | SmartCare | PSV | interruptions (S); duration of ventilation, duration of weaning, ICU LOS, VFD–28 (E–cy) |
| Lal *et al.* (2015) | [70] | crossover | 27 | preterm | 2 x 12 hours | Avea–CliO_2_ | manual FiO_2_ control | time within unsafe SpO_2_ ranges (S); manual adjustments (W) |
| Kaam *et al.* (2015) ^++^ | [71] | crossover | 80 | preterm | 2 x 24 hours | Avea–CliO_2_ | manual FiO_2_ control | time within unsafe SpO_2_ ranges (S); manual adjustments (W) |
| Kallio *et al.* (2015) | [72] | parallel | 170 | critically ill paediatric | 3.3 hours | NAVA | PCV | V_T_, FiO_2_, (E–ness); AE (S); duration of ventilation, ICU LOS (E–cy) |
| Soydan *et al.* (2022) | [73] | crossover | 30 | critically ill paediatric | 2 x 2 hours | ASV with closed loop FiO_2_ | ASV with manual FiO_2_ | time within unsafe SpO_2_ ranges (S); manual adjustments (W) |
| Abbreviations: min: minutes; ASV: Adaptive Support Ventilation; SIMV: synchronized intermitted mandatory ventilation: PSV: pressure support ventilation; FiO_2_: fraction of inspired oxygen; NAVA: neurally adjusted ventilatory assist; PAV+: proportional assist ventilation, ABG: arterial blood gas; ARF: acute respiratory failure; AHRF: hypoxemic respiratory failure;  (E–ness) = effectiveness; (S) = safety; (E–cy) = efficacy; (W) = workload  Reported data regarding duration refers to the duration in the closed loop ventilation group  ^+^ abstract available only for this study; ^++^ in this study a part of the included patients received both non–invasive and invasive ventilation | | | | | | | | |

| **eTable S2.** Fragility index and loss to follow–up | | | | | | |
| --- | --- | --- | --- | --- | --- | --- |
| **first author (year)** | **ref** | **closed loop mode** | **primary endpoint** | **result** | **FI** | **loss to follow–up (n)** |
| Xirouchaki *et al.* (2008) | [27] | PAV+ | failure rate | 11.1% *vs* 22.0% (p = 0.040) | 1 | 0 |
| Elganady *et al.* (2014) | [41] | PAV+ | weaning success | 90% vs 66.7% (p = 0.028) | 0 | 0 |
| Moradian *et al.* (2017) | [52] | ASV | incidence of atelectasis | 35.3 vs 64.7% (p = 0.04) | 1 | 8 |
| de Bie *et al.* (2020) | [57] | ASV | proportion of time in predefined optimal ventilatory zones | 55.2% *vs* 25.5%  (95% CI 22.1–37.4, p < 0.001) | 18 | 0 |
| Hadfield *et al.* (2020) | [61] | NAVA | compliance (>65% adherence to assigned mode) | 66.7% (95% CI 50.3–80.0%) *vs* 100% (95% CI 89.0–100.0%) | 7 | 1 |
| Abbreviations: PAV+: proportional assist ventilation; ASV: adaptive support ventilation; NAVA: neurally adjusted ventilatory assist; CI: confidence interval; FI: fragility index  Closed loop ventilation values are presented first. Data are presented as percentages with 95% confidence interval if available. | | | | | | |

| **eTable S3.** Effectiveness of closed loop ventilation | | | | | |  | | |
| --- | --- | --- | --- | --- | --- | --- | --- | --- |
| **first author (year)** | **ref** | | **closed loop mode** | | **results (endpoint)** | ***P*** | | |
| Xirouchaki *et al.* (2008) | [27] | | PAV+ | | 6.6 [6.0–7.1] *vs* 6.7 [6.0–7.4] ml kg^-1^ PBW (V_T_) | NS | | |
| Dongelmans *et al.* (2009) | [28] | | ASV | | 8.6 ± 0.8 *vs* 7.1 ± 1.4 ml kg^-1^ PBW (V_T_) | NS | | |
| Coisel *et al.* (2010) | [31] | | NAVA | | 410 [371–457] *vs* 463 [394–502] ml (V_T_) | < 0.01 | | |
| Kirakli *et al.* (2011) | [32] | | ASV | | 5.7 [5.3–6.3] *vs* 5.4 [4.9–6.9] ml kg^-1^ PBW (V_T_) | NS | | |
| Arnal *et al.* (2012) | [33] | | INTELLiVENT–ASV | | 8.1 [7.7–8.6] *vs* 8.3 [7.8–9.0] ml kg^-1^ PBW (V_T_) | < 0.01 | | |
|  |  |  |  |  | 30 [30–39] *vs* 40 [30–50] % (FiO_2_) | < 0.01 | | |
| Argawal *et al.* (2013) | [35] | | ASV | | 5.6 ± 0.8 *vs* 6.6 ± 1.4 ml kg^-1^ PBW (V_T_) | < 0.01 | | |
| Clavieras *et al.* (2013) | [37] | | INTELLiVENT–ASV | | 8.4 [7.9–8.6] *vs* 7.6 [6.6–9.0] ml kg^-1^ PBW (V_T_) | < 0.05 | | |
|  |  |  |  |  | 31 [30–32] *vs* 33 [30–41] % (FiO_2_) | NS | | |
| Lellouche *et al.* (2013) | [38] | | INTELLiVENT–ASV | | 7.8 ± 0.5 *vs* 10.1 ± 1.3 ml kg^-1^ PBW (V_T_) | < 0.01 | | |
|  |  |  |  |  | 33 ± 6 vs 47 ± 10 % (FiO_2_) | < 0.01 | | |
| Taniguchi *et al.* (2015) | [44] | | SmartCare | | 500 [450–630] *vs* 500 [420–600] ml (V_T_) | NS | | |
|  |  |  |  |  | 0.30 (0.25–0.30) *vs* 0.30 (0.25–0.30) (FiO_2_) | NS | | |
| Zhu *et al.* (2015) | [46] | | ASV | | 8.6 ± 1.2 vs 9.0 ± 2.0 ml kg^-1^ PBW (V_T_) | NS | | |
|  |  |  |  |  | 42 ± 6 *vs* 43 ± 5 % (FiO_2_) | NS | | |
| Bialais *et al.* (2016) | [47] | | INTELLiVENT–ASV | | 7.9 [7.6–8.2] *vs* 7.5 [7.1–8.1] ml kg^-1^ PBW (V_T_) | NS | | |
|  |  |  |  |  | 33 [34–42] vs 36 [33–44] % (FiO_2_) | NS | | |
| Bosma *et al.* (2016) | [48] | | PAV+ | | 9.6 ± 3.0 *vs* 10.0 ± 2.5 ml kg^-1^ PBW (V_T_) | NS | | |
|  |  |  |  |  | 10.8 ± 3.8 *vs* 12.3 ± 5.4 cm H_2_O (ΔP) | p < 0.01 | | |
| Arnal *et al.* (2018) | [53] | | INTELLiVENT–ASV | | 8.0 [7.0–8.1] *vs* 7.1 [7.0–9.0] ml kg^-1^ PBW (V_T_) | NS | | |
|  |  |  |  |  | 35 [32–42] *vs* 36 [32–44] % (FiO_2_) | NS | | |
| Botha *et al.* (2018) | [54] | | PAV+ | | 491 [402–634] *vs* 511 [417–641] ml (V_T_) | NS | | |
| Grieco *et al.* (2018) | [55] | | SmartCare | | 7.2 [6.2–8.3] *vs* 8.2 [7.1–9.1] ml kg^-1^ PBW (V_T_) | < 0.05 | | |
| de Bie *et al.* (2020) | [57] | | INTELLiVENT–ASV | | 6.4 [5.8–6.6] *vs* 7.8 [7.7–8] ml kg^-1^ PBW (V_T_) | < 0.01 | | |
|  |  |  |  |  | 10.6 ± 2.6 *vs* 11.5 ± 2.3 cm H_2_O (ΔP) | < 0.01 | | |
|  |  |  |  |  | 6.9 ± 2.6 *vs* 9.1 ± 2.3 J min^-1^ (MP) | < 0.01 | | |
|  |  |  |  |  | 33 [32–44] *vs* 43 [41–52] % (FiO_2_) | < 0.01 | | |
| Chelly *et al.* (2020) | [58] | | INTELLiVENT–ASV | | 10 ± 2 *vs* 10 ± 3 ml kg^-1^ PBW (V_T_) | NS | | |
| Diniz–Silva *et al.* (2020) | [59] | | NAVA | | 5.8 ± 1.1 *vs* 5.6 ± 1.0 ml kg^-1^ PBW (V_T_) | NS | | |
| Eremenko *et al.* (2020) | [60] | | ASV | | 7.0 [6–8.5] *vs* 7 [6–10] ml kg^-1^ PBW (V_T_) | NS | | |
| Cammarota *et al.* (2022) | [64] | | NAVA | | 6.3 [5.3–7.4] *vs* 5.6 [4.7–7.4] ml kg^-1^ PBW (V_T_) | < 0.05 | | |
|  |  |  |  |  | 7.7 [5.1–8.1] *vs* 6.6 [6.1–7.8] cm H_2_O (ΔP) | < 0.01 | | |
|  |  |  |  |  | 15.5 [14.0–21.8] *vs* 16.5 [13.5–18.8] J min^-1^ (MP) | NS | | |
| Baedorf Kassis *et al.* (2022) | [65] | | ASV | | 6.29 [5.87–6.99] *vs* 6.04 [6.01–6.06] ml kg^-1^ PBW (V_T_) | < 0.05 | | |
|  |  |  |  |  | 11.7 [10.7–15.1] *vs* 12.8 [9.0–15.8] cm H_2_O (ΔP) | NS | | |
|  |  |  |  |  | 28.2 [22.2–36.4] *vs* 26.9 [23.8–37.9] J min^-1^ (MP) | NS | | |
| Abbreviations: V_T_: tidal volume; ASV: Adaptive Support Ventilation; C_RS_: compliance of the respiratory system; ΔP: driving pressure; MP: mechanical power; I–ASV: INTELLiVENT–ASV; FiO_2_: fraction of inspired oxygen; SpO_2_: peripheral pulse oximetry; NAVA: neurally adjusted ventilatory assist; PAV+: proportional assist ventilation.  Closed loop ventilation values are presented first. Data are presented as mean ± standard deviation or median [interquartile range]. | | | | | | | | |
| **eTable S4**. Safety of closed loop ventilation | | | | | | |  |  |
| **first author (year)** | **ref** | **closed loop mode** | | **results** | | | ***P*** |  |
| Petter *et al.* (2003) | [24] | ASV | | high Pinsp 2/18 (15 %) *vs* 11/16 (85 %) | | | < 0.01 |  |
| Xirouchaki *et al.* (2008) | [27] | PAV+ | | adverse events 0/0 (0 %) *vs* 0/0 (0 %) | | | NS |  |
| Stahl *et al.* (2009) | [29] | SmartCare | | reintubations 8/26 (30.8 %) *vs* 6/26 (23.1 %) | | | NS |  |
| Coisel *et al.* (2010) | [31] | NAVA | | proportion of time in V_T_ <5 ml kg^-1^ PBW 5.1 [3.6–17.8] *vs* 0.4 [0–1.5] % | | | < 0.01 |  |
| Arnal *et al.* (2012) | [33] | INTELLiVENT–ASV | | adverse events 0/0 (0 %) *vs* 0/0 (0 %) | | | NS |  |
| Burns *et al.* (2013) | [36] | SmartCare | | reintubations 9/49 (18.4 %) *vs* 11/43 (25.6 %) | | | NS |  |
| Jouvet *et al.* (2013) | [69] | SmartCare | | interruptions 3 [0–18] *vs* 0 [0–0] | | | NR |  |
| Liu *et al.* (2013) | [39] | SmartCare | | reintubations 3 (15.8 %) *vs* 4 (20 %) | | | NS |  |
| Celli *et al.* (2014) | [40] | ASV | | episodes of Ppeak >35 cmH_2_O 0.8 ± 2.2 *vs* 2.5 ± 3 | | | NS |  |
|  |  |  |  | number of patients with episodes of Ppeak > 35 cm H_2_O 2/10 (20 %) *vs* 7/10 (70 %) | | | NR |  |
| Mohamed *et al.* (2014) | [42] | ASV | | reintubations 3/25 (12 %) *vs* 4/25 (16 %) | | | NS |  |
| Taniguchi *et al.* (2015) | [44] | SmartCare | | reintubations 2/35 (5.7 %) *vs* 2/35 (5.7 %) | | | NS |  |
|  |  |  |  | ventilator related malfunction 3/35 (8.6 %) *vs* 0/35 (0 %) | | | NS |  |
| Zhu *et al.* (2015) | [46] | ASV | | reintubations 2/30 (8.0 %) *vs* 0/31 (0.0 %) | | | NS |  |
| Bosma *et al.* (2016) | [48] | PAV+ | | adverse events 0/0 (0 %) *vs* 0/0 (0 %) | | | NS |  |
| Moradian et al. (2017) | [52] | ASV | | incidence of atelectasis  12/34 (35.3 %) vs 22/34 (64.7 %) | | | <0.05 |  |
| Botha *et al.* (2018) | [54] | PAV+ | | reintubations 1/24 (4.2 %) *vs* 2/24 (8.3 %) | | | NS |  |
| Delgado *et al.* (2018) | [56] | PAV+ | | failure 22/52 (42 %) *vs* 0/50 (0 %) | | | NS |  |
| De Bie *et al.* (2020) | [57] | INTELLiVENT–ASV | | breaths with SpO2 <85 % 229/558.459 (0.0 %) *vs* 1.123/697.352 (0.2 %) | | | < 0.01 |  |
| Chelly *et al.* (2020) | [58] | INTELLiVENT–ASV | | episodes of SpO_2_ <90 % 30/265 (11 %) *vs* 50/265 (19 %) | | | < 0.05 |  |
|  |  |  |  | adverse events 0/0 (0 %) *vs* 0/0 (0 %) | | | NS |  |
| Eremenko *et al.* (2020) | [60] | ASV | | reintubations 0/40 (0 %) *vs* 0/38 (0 %) | | | NS |  |
| Kacmarek *et al.* (2020) | [62] | NAVA | | reintubations 17/153 (11.1 %) *vs* 33/153 (21.6 %) | | | < 0.05 |  |
| Baedorf Kassis *et al.* (2022) | [65] | ASV | | adverse events 0/0 (0 %) *vs* 0/0 (0 %) | | | NS |  |
| Seghal *et al*. (2022) | [66] | ASV | | post–extubation respiratory failure 2/24 (8.3 %) *vs* 1/24 (4.2 %) | | | NS |  |
|  |  |  |  | ventilator–associated pneumonia 2/24 (8.3 %) *vs* 1/24(4.2 %) | | | NS |  |
|  |  |  |  | barotrauma 0/24 (0 %) *vs* 0/24 (0 %) | | | NR |  |
| Claure *et al.* (2011) | [68] | automated FiO_2_ control | | episodes of SpO_2_ <87 % 61 ±19 *vs* 77 ±18 seconds | | | < 0.05 |  |
|  |  |  |  | adverse events 0/0 (0 %) *vs* 0/0 (0 %) | | | NS |  |
| Lal *et al.* (2015) | [70] | automated FiO_2_ control | | proportion of time SpO_2_ <90 % 18.1 [12.7–23.6] *vs* 25.9 [17.8–30.7] % | | | < 0.05 |  |
| Kaam *et al.* (2015) | [71] | automated FiO_2_ control | | proportion of time in target SpO_2_ range 62 ±17 *vs* 54 ±16 % | | | < 0.01 |  |
| Kallio *et al.* (2015) | [72] | NAVA | | 14/85 (16 %) vs 19/85 (22 %) AE | | | NS |  |
| Soydan *et al.* (2022) | [73] | ASV with closed loop FiO_2_ | | proportion of time in target SpO_2_ range 96 [93–99] *vs* 78 [51–98] % | | | < 0.01 |  |
| Abbreviations: ASV: Adaptive Support Ventilation; PCV: pressure controlled ventilation; FiO_2_: fraction of inspired oxygen; NAVA: neurally adjusted ventilatory assist; PAV+: proportional assist ventilation; FiO_2_: fraction of inspired oxygen; SpO_2_: peripheral pulse oximetry; ARF: acute respiratory failure; Pinsp: inspiratory pressure; Ppeak: peak airway pressure; NR: not reported; AE adverse events.  Closed loop ventilation values are presented first. Data are presented as mean ± standard deviation, median [interquartile range] or N (%). | | | | | | | |  |

| **eTable S5**. Efficacy of closed loop ventilation | | | |  |
| --- | --- | --- | --- | --- |
| **first author (year)** | **ref** | **closed loop mode** | **results (endpoint)** | ***P*** |
| Sulzer *et al.* (2001) | [23] | ASV | 193 [149–273] *vs* 243 [186–516] minutes (ventilation duration) | < 0.05 |
| Petter *et al.* (2003) | [24] | ASV | 2.7 [2.1–4.2] *vs* 3.2 [2.7–4.0] hours (ventilation duration) | NS |
| Jiang *et al.* (2006) | [25] | SmartCare | 8.54 ± 2.09 *vs* 13.32 ± 2.19 days (ventilation duration) | NS |
|  |  |  | 77% *vs* 40 % (weaning success at day 7) | < 0.05 |
| Rose *et al.* (2008) | [26] | SmartCare | 43 [6–169] *vs* 40 [14–87] hours (time to extubation) | NS |
| Dongelmans *et al.* (2009) | [28] | ASV | 16.4 [12.5–20.8] *vs* 16.3 [13.7–19.3] hours (ventilation duration) | NS |
| Stahl *et al.* (2009) | [29] | SmartCare | 0.64 [0.28–5.80] *vs* 2.33 [0.50–6.42] days (weaning duration) | NS |
|  |  |  | 21.78 [10.79–30.60] *vs* 19.65 [11.33–30.39] days (ICU LOS) | NS |
| Ma *et al.* (2010) | [30] | SmartCare | 254 ± 96 *vs* 502 ± 9 hours (weaning duration) | < 0.05 |
|  |  |  | 7.3 ± 1.9 *vs* 14.6 ± 1.7 days (ICU LOS) | < 0.05 |
| Kirakli *et al.* (2011) | [32] | ASV | 24 [20–62] *vs* 72 [24–169] hours (weaning duration) | < 0.05 |
| Schädler *et al.* (2012) | [34] | SmartCare | 10 [3–60] v*s* 17 [3–85] hours (weaning duration) | NS |
|  |  |  | 31 [19–101] *vs* 39 [20–118] hours (ventilation duration) | NS |
|  |  |  | 3.8 [1.3–8.2] v*s* 3.4 [1.3–11.5] days (ICU LOS) | NS |
| Agarwal *et al.* (2013) | [35] | ASV | 6 [3.5–11.5] *vs* 5 [3–11] days (ventilation duration) | NS |
|  |  |  | 9 [4.5–15.5] *vs* 8 [6–14] days (ICU LOS) | NS |
|  |  |  | 11 [6.5–18.5] *vs* 11 [8–16] days (hospital LOS) | NS |
|  |  |  | 8/23 (34.7 %) *vs* 9/25 (36 %) (hospital mortality) | NS |
| Burns *et al.* (2013) | [36] | SmartCare | 4.0 [2.0–7.0] *vs* 5.0 [3.0–19.0] days (weaning duration) | 0.01 |
|  |  |  | 10.5 [8.0–18.0] *vs* 11.0 [6.0–25.0] days (ventilation duration) | NS |
|  |  |  | 16.0 [12.0–20.0] vs 18.5 [10.0–33.0] days (ICU LOS survivors) | NS |
|  |  |  | 28.0 [20.0–44.0] *vs* 43.0 [20.0–55.0] days (hospital LOS survivors) | NS |
|  |  |  | 13/49 (26.5 %) *vs* 11/43 (25.6 %) (hospital mortality) | NS |
| Jouvet *et al.* (2013) | [69] | SmartCare | 36 ± 36 *vs* 142 ± 150 hours (weaning duration) | <0.01 |
|  |  |  | 200 ± 186 *vs* 288 ± 206 hours (ventilation duration) | NS |
|  |  |  | 24.5 ± 7.0 *vs* 21.9 ± 6.2 days (VFD–28) | NS |
|  |  |  | 9 ± 5 *vs* 17 ± 14 days (ICU LOS) | NS |
| Lellouche *et al.* (2013) | [38] | INTELLiVENT–ASV | 5.2 [4.4–7.4] *vs* 6.6 [4.8–8.7] days (ventilation duration) | NS |
| Liu *et al.* (2013) | [39] | SmartCare | 29.0 [26.3–45.8] *vs* 45.5 [27.8–99.0] hours (weaning duration) | <0.05 |
|  |  |  | 138 [82.0–166.5] *vs* 194.5 [76.5–243.5] hours (ventilation duration) | NS |
|  |  |  | 14.0 [10.0–33.5] *vs* 28.5 [13.8–63.5] days (ICU LOS) | NS |
|  |  |  | 4/19 (21.1 %) vs 4/20 (20.0 %) (ICU mortality) | NS |
| Celli *et al.* (2014) | [40] | ASV | 90 ± 13 *vs* 153 ± 22 minutes (ventilation duration) | 0.05 |
| Elganady *et al.* (2014) | [41] | PAV+ | success group: 2.43 ± 0.91 *vs* 3.85 ± 1.23 days(ventilation duration) | <0.01 |
|  |  |  | failure group: 6.33 ± 0.58 *vs* 8.90 ± 0.88 days (ventilation duration) | <0.05 |
|  |  |  | 90 % vs 66.7 % (weaning success) | <0.05 |
|  |  |  | success group: 3.70 ± 0.94 *vs* 5.45 ± 1.43 days (ICU LOS) | <0.01 |
|  |  |  | failure group: 8.33 ± 0.58 *vs* 10.0 ± 1.05 days (ICU LOS) | <0.05 |
|  |  |  | success group: 4.81 ±1.24 *vs* 6.65 ± 1.57 days (hospital LOS) | <0.01 |
|  |  |  | failure group: 9.67 ± 0.58 *vs* 11.50 ± 1.60 days (hospital LOS) | <0.05 |
|  |  |  | 1/30 (3.3 %) *vs* 2/30 (6.7 %) (28-day mortality) | NS |
| Mohamed *et al.* (2014) | [42] | ASV | 113 ± 22.1 159 ± 37.2 hours (ventilation duration) | < 0.05 |
|  |  |  | 7.3 ± 2.6 *vs* 11.7 ± 4.2 days (ICU LOS) | < 0.05 |
|  |  |  | 1/25 (4 %) *vs* 2/25 (8 %) (28–day mortality) | NS |
| Kirakli *et al.* (2015) | [43] | ASV | 4 [2–6] *vs* 4 [3–9] days (ventilation duration) | < 0.05 |
|  |  |  | 64/114 (56 %) *vs* 54/115 (47 %) (weaning success) | NS |
|  |  |  | 46/114 (40 %) *vs* 48/115 (42 %) (28–day mortality) | NS |
| Taniguchi *et al.* (2015) | [44] | SmartCare | 4.1 [2.7–7.1] *vs* 3.5 [2.0–7.3] days (ventilation duration) | NS |
|  |  |  | 110.0 [80.0–130] *vs* 60.0 [50.0–80.0] minutes (weaning duration) | < 0.01 |
| Teixeira *et al.* (2015) | [45] | PAV+ | 11.5 ± 8.9 *vs* 11.9 ± 7.4 days (ICU LOS) | NS |
| Zhu *et al.* (2015) | [46] | ASV | 295 [196–413] *vs* 421 [297–653] minutes (ventilation duration) | < 0.05 |
|  |  |  | 21 [18–22] *vs* 22 [18–23] hours (ICU LOS) | NS |
|  |  |  | 10.5 [9–14.25] *vs* 11 [9–16] days (hospital LOS) | NS |
| Bialais *et al.* (2016) | [47] | INTELLiVENT–ASV | 5.5 [6.0–13.0] *vs* 8.0 [6.5–15.3] days (ventilation duration) | NS |
|  |  |  | 11.5 [10.8–20.8] *vs* 13.0 [11.6–24.3] days (ICU LOS) | NS |
|  |  |  | 1 (3 %) *vs* 5 (18 %) (mortality) | < 0.05 |
| Bosma *et al.* (2016) | [48] | PAV+ | 3.9 [2.8–8.4] *vs* 4.9 [2.9–26.3] days (time to successful extubation) | NS |
|  |  |  | 7.3 [5.2–11.4] *vs* 12.4 [7.5–30.8] days (time to live ICU discharge) | < 0.05 |
| Demoule *et al.* (2016) | [49] | NAVA | 10.0 [6.0–17.0] *vs* 10.0 [7.0–16.0] days (ventilation duration) | NS |
| Yazdannik *et al.* (2016) | [50] | ASV | 4.83 *vs* 6.71 hours (ventilation duration) | < 0.01 |
|  |  |  | 140.6 *vs*145.1 hours (hospital LOS) | < 0.01 |
| Fot *et al.* (2017) | [51] | INTELLiVENT–ASV | 3.2 [1.9–5.2] *vs* 3.3 [2.6–4.2] days (weaning duration) | NS |
|  |  |  | 2 [1.0–3.0] *vs* 1 [1.0–3.0] days (ICU LOS) | NS |
| Moradian *et al.* (2017) | [52] | ASV | 29 ± 169 *vs* 348 ± 150 minutes (ventilation duration) | NS |
|  |  |  | 33.93 ± 9.24 *vs* 36.29 ± 10.71 hours (ICU LOS) | NS |
|  |  |  | 6 ± 1.45 *vs* 6.69 ± 2.04 days (hospital LOS) | < 0.01 |
| Arnal *et al.* (2018) | [53] | INTELLiVENT–ASV | 6.0 [3.0–9.0] *vs* 6.5 [4.0–14.5] days (ventilation duration) | NS |
|  |  |  | 4.0 [1.0–5.5] *vs* 2.0 [1.0–4.0] days (ICU LOS) | NS |
| Botha *et al.* (2018) | [54] | PAV+ | 84.3 [25.7–244.8] *vs* 135.9 [49.1–301.0] days (weaning duration) | NS |
|  |  |  | 1/25 (4.0 %) *vs* 6/24 (25.0 %) (mortality) | < 0.05 |
| Delgado *et al.* (2018) | [56] | PAV+ | 3 *vs* 3 days (duration of ventilation) | NS |
|  |  |  | 9 *vs* 8 days (ICU LOS) | NS |
| de Bie *et al.* (2020) | [57] | INTELLiVENT–ASV | 0.20 [0.14–0.30] *vs* 0.21 [0.14–0.33] days (ventilation duration) | NS |
|  |  |  | 0.3 [0.3–0.6] *vs* 0.4 [0.3–0.7] days (ICU LOS) | NS |
| Eremenko *et al.* (2020) | [60] | ASV | 172 ± 71 *vs* 187 ± 72 minutes (ventilation duration) | NS |
|  |  |  | 1[1–1] *vs* 1[1–1] days (ICU LOS) | NS |
|  |  |  | 0/40 (0 %) *vs* 0/38 (0 %) (hospital mortality) | NS |
| Hadfield *et al.* (2020) | [61] | NAVA | 15.5 [0.0–23.0] *vs* 0.0 [0.0–20.5] days (VFD day 28) | < 0.05 |
|  |  |  | 9.1 [6.0–21.9] *vs* 14.8 [7.0–33.1] days (ICU LOS) | NS |
| Kacmarek *et al.* (2020) | [62] | NAVA | 22 [3–25] *vs* 18 [0–24] days (VFD day 28) | < 0.05 |
| Liu *et al.* (2020) | [63] | NAVA | 3.0 [1.2 to 8] *vs* 7.4 [2–28] days (weaning duration) | < 0.01 |
| Sehgal *et al.* (2022) | [66] | ASV | 90.4 ± 87.3 vs 97 ± 112 hours (ventilation duration) | NS |
|  |  |  | 4.8 ± 3.8 *vs* 5.5 ± 4.9 days (ICU LOS) | NS |
|  |  |  | 4.9 ± 3.8 *vs* 5.7 ± 4.9 days (hospital LOS) | NS |
|  |  |  | 0/24 (0 %) *vs* 0/24 (0 %) (mortality) | NS |
| Zhang et al. (2022) | [67] | ASV | 258.9 ± 25.6 *vs* 302.2 ± 29.6 hours (weaning duration) | < 0.01 |
|  |  |  | 11.19 ± 3.15 vs 15.26 ± 3.02 days (ICU LOS) | < 0.01 |
| Kallio *et al.* (2015) | [72] | NAVA | 3.3 *vs* 6.6 hours (ventilation duration) | NS |
|  |  |  | 49.5 *vs* 72.8 hours (ICU LOS) | NS |
| Abbreviations: ASV: Adaptive Support Ventilation; PCV: pressure controlled ventilation; ICU: intensive care unit; LOS: length of stay; FiO_2_: fraction of inspired oxygen; NAVA: neurally adjusted ventilatory assist; PAV+: proportional assist ventilation; VFD: ventilator free days  Closed loop ventilation values are presented first. Data are presented as mean ± standard deviation or median [interquartile range]. | | | | |

| **eTable S6.** Workloads with closed loop ventilation | | | |  |
| --- | --- | --- | --- | --- |
| **authors** | **ref** | **closed loop mode** | **result (endpoint)** | ***P*** |
| Petter *et al.* (2003) | [24] | ASV | 2.4 ± 0.7 *vs* 4.0 ± 0.8 per patient (manual adjustments) | < 0.05 |
|  |  |  | 0.7 ± 2.4 *vs* 2.9 ± 3.0 per patient (alarms) | < 0.05 |
| Jiang *et al.* (2006) | [25] | SmartCare | 3.5 ± 3.1 *vs* 6.6 ± 3.7 per patient (ABG analyses) | < 0.05 |
| Stahl *et al.* (2009) | [29] | SmartCare | 0.0 [0.0–0.0] *vs* 0.15 [0.11–0.27] per hour (manual adjustments) | < 0.01 |
| Ma *et al.* (2010) | [30] | SmartCare | 5 ± 1 *vs* 13 ± 3 (manual adjustments) | < 0.05 |
| Lellouche *et al.* (2013) | [38] | INTELLiVENT–ASV | 5 *vs* 148 per 4 hours (manual adjustments) | < 0.01 |
| Kirakli *et al.* (2015) | [43] | ASV | (2 [1–2] *vs* 3 [2–5] (manual adjustments) | < 0.01 |
| Bialais *et al.* (2016) | [47] | INTELLiVENT–ASV | 3 ± 4 *vs* 13 ± 11 per 48 hours (manual adjustments) | < 0.01 |
| Fot *et al.* (2017) | [51] | INTELLiVENT–ASV | 0 *vs* 7 [5–9] per patient (manual adjustments) | < 0.01 |
| Moradian *et al.* (2017) | [52] | ASV | 5.66 ± 2.07 *vs* 8.06 ± 2.91) (manual adjustments) | NR |
|  |  |  | 10.24 ± 3.42 vs. 14.53 ± 4.81 (alarms) |  |
| Arnal *et al.* (2018) | [53] | INTELLiVENT–ASV | 5 [4–7] *vs* 10 [7–17] per patient per 24 hours (manual adjustments) | < 0.01 |
| Chelly *et al.* (2020) | [58] | INTELLiVENT–ASV | 41 (15%) vs. 69 (26%) per period (manual adjustments) | < 0.05 |
| Claure *et al.* (2011) | [68] | automated FiO_2_ control | 10 ± 9 *vs* 112 ± 59 per 24 hours (manual adjustments) | < 0.01 |
| Celli *et al.* (2014) | [40] | ASV | 1.5 ± 1 *vs* 6 ± 2 (manual adjustments) | <0.01 |
| Lal *et al.* (2015) | [70] | automated FiO_2_ control | 0 [0–2] *vs* 63 [26–107] per 12 hours (manual adjustments) | < 0.01 |
| Kaam *et al.* (2015) | [71] | automated FiO_2_ control | 1 [0–3] *vs* 102 [73–173] (manual adjustments) | < 0.01 |
| Eremenko *et al.* (2020) | [60] | ASV | 2 [1–4] *vs* 4 [2–6] per patient (manual adjustments) | < 0.01 |
|  |  |  | 3 [2–5] vs 4 [2–6] per patient (approaches to the ventilator) | < 0.01 |
|  |  |  | 99 ± 35 *vs* 166 ± 70 seconds per patient (time spent at the ventilator) | < 0.01 |
| Soydan *et al.* (2022) | [73] | ASV with closed loop FiO_2_ | 52 [11.8–67] *vs* 1 [0–2] per 2 hours (automated adjustments) | < 0.01 |
| Abbreviations: ABG: arterial blood gas; ASV: Adaptive Support Ventilation; NAVA: neurally adjusted ventilatory assist; PAV+: proportional assist ventilation; NR: not reported.  Closed loop ventilation values are presented first. Data are presented as mean ± standard deviation or median [interquartile range]. | | | | |

**
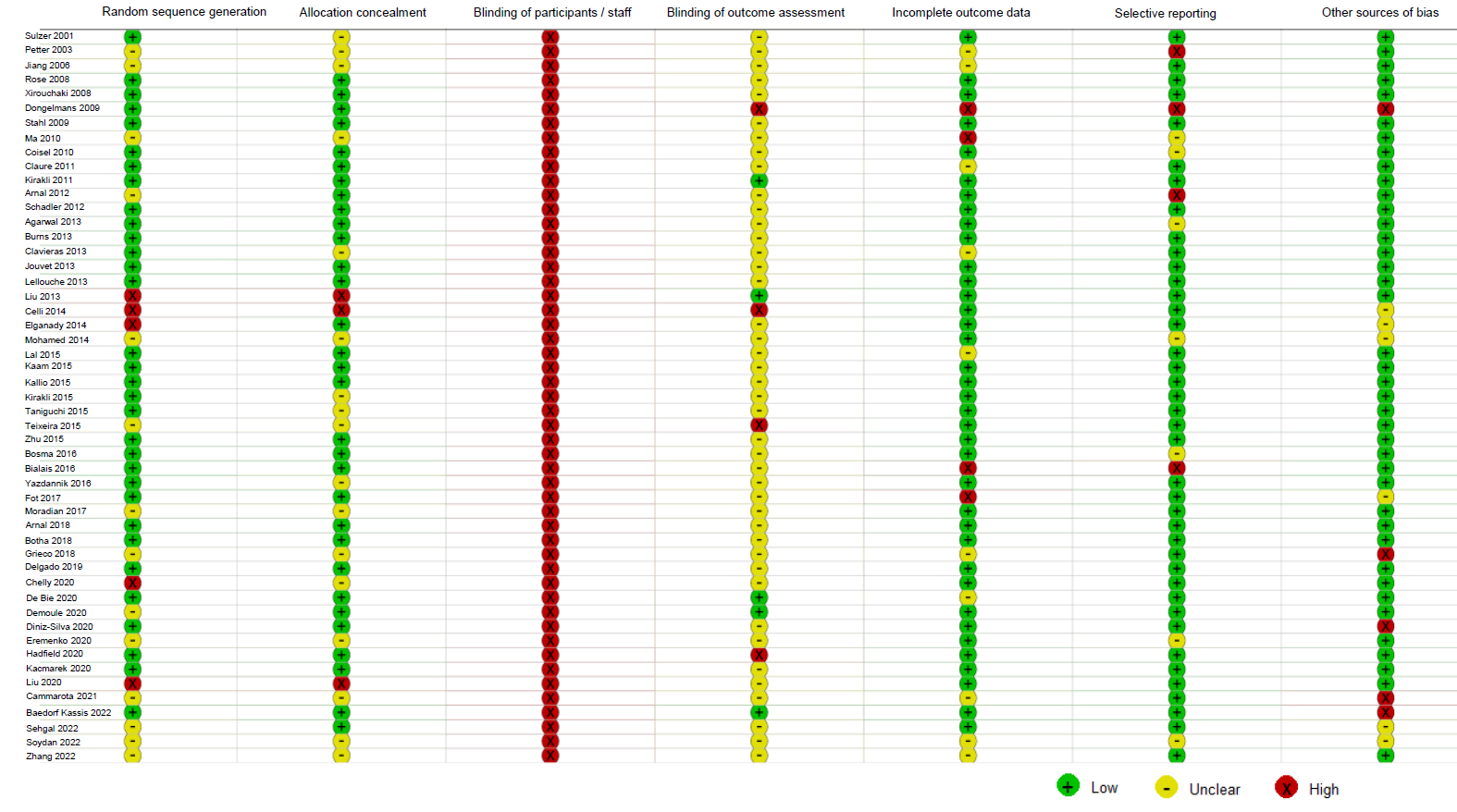
 eFigure S1.** Assessment risk of bias

**Search details**

Search:

"Interactive Ventilatory Support"[Mesh] OR ("Respiration, Artificial"[Mesh] AND "Automation"[Mesh]) OR "closed-loop ventilation" OR "closed loop ventilation" OR "automated ventilation" OR ("mechanical ventilation" AND "explicit computerized protocols")

Filters:

Language: English

Age: Adult 19+ years + all filters from birth – 18 years

Last literature search at 10-01-2023

**References**

1. Brower RG, Matthay MA, Morris A, Schoenfeld D, Thompson BT, Wheeler A. Ventilation with lower tidal volumes as compared with traditional tidal volumes for acute lung injury and the acute respiratory distress syndrome. N Engl J Med. 2000;342(18):1301-8.

2. Costa ELV, Slutsky AS, Brochard LJ, Brower R, Serpa-Neto A, Cavalcanti AB, et al. Ventilatory Variables and Mechanical Power in Patients with Acute Respiratory Distress Syndrome. Am J Respir Crit Care Med. 2021;204(3):303-11.

3. Esteban A, Frutos-Vivar F, Muriel A, Ferguson ND, Peñuelas O, Abraira V, et al. Evolution of mortality over time in patients receiving mechanical ventilation. Am J Respir Crit Care Med. 2013;188(2):220-30.

4. Simonis FD, Serpa Neto A, Binnekade JM, Braber A, Bruin KCM, Determann RM, et al. Effect of a Low vs Intermediate Tidal Volume Strategy on Ventilator-Free Days in Intensive Care Unit Patients Without ARDS: A Randomized Clinical Trial. Jama. 2018;320(18):1872-80.

5. Amato MB, Meade MO, Slutsky AS, Brochard L, Costa EL, Schoenfeld DA, et al. Driving pressure and survival in the acute respiratory distress syndrome. N Engl J Med. 2015;372(8):747-55.

6. Aoyama H, Pettenuzzo T, Aoyama K, Pinto R, Englesakis M, Fan E. Association of Driving Pressure With Mortality Among Ventilated Patients With Acute Respiratory Distress Syndrome: A Systematic Review and Meta-Analysis. Crit Care Med. 2018;46(2):300-6.

7. Pereira SM, Tucci MR, Morais CCA, Simões CM, Tonelotto BFF, Pompeo MS, et al. Individual Positive End-expiratory Pressure Settings Optimize Intraoperative Mechanical Ventilation and Reduce Postoperative Atelectasis. Anesthesiology. 2018;129(6):1070-81.

8. Pelosi P, Ball L, Barbas CSV, Bellomo R, Burns KEA, Einav S, et al. Personalized mechanical ventilation in acute respiratory distress syndrome. Crit Care. 2021;25(1):250.

9. Gattinoni L, Tonetti T, Cressoni M, Cadringher P, Herrmann P, Moerer O, et al. Ventilator-related causes of lung injury: the mechanical power. Intensive Care Med. 2016;42(10):1567-75.

10. Cressoni M, Gotti M, Chiurazzi C, Massari D, Algieri I, Amini M, et al. Mechanical Power and Development of Ventilator-induced Lung Injury. Anesthesiology. 2016;124(5):1100-8.

11. Protti A, Andreis DT, Monti M, Santini A, Sparacino CC, Langer T, et al. Lung stress and strain during mechanical ventilation: any difference between statics and dynamics? Crit Care Med. 2013;41(4):1046-55.

12. Serpa Neto A, Amato MBP, Schultz MJ. Dissipated Energy is a Key Mediator of VILI: Rationale for Using Low Driving Pressures. In: Vincent J-L, editor. Annual Update in Intensive Care and Emergency Medicine 2016. Cham: Springer International Publishing; 2016. p. 311-21.

13. Serpa Neto A, Deliberato RO, Johnson AEW, Bos LD, Amorim P, Pereira SM, et al. Mechanical power of ventilation is associated with mortality in critically ill patients: an analysis of patients in two observational cohorts. Intensive Care Med. 2018;44(11):1914-22.

14. van Meenen DMP, Algera AG, Schuijt MTU, Simonis FD, van der Hoeven SM, Neto AS, et al. Effect of mechanical power on mortality in invasively ventilated ICU patients without the acute respiratory distress syndrome: An analysis of three randomised clinical trials. Eur J Anaesthesiol. 2022.

15. Urner M, Jüni P, Hansen B, Wettstein MS, Ferguson ND, Fan E. Time-varying intensity of mechanical ventilation and mortality in patients with acute respiratory failure: a registry-based, prospective cohort study. Lancet Respir Med. 2020;8(9):905-13.

16. Helmerhorst HJ, Schultz MJ, van der Voort PH, de Jonge E, van Westerloo DJ. Bench-to-bedside review: the effects of hyperoxia during critical illness. Crit Care. 2015;19(1):284.

17. Girardis M, Busani S, Damiani E, Donati A, Rinaldi L, Marudi A, et al. Effect of Conservative vs Conventional Oxygen Therapy on Mortality Among Patients in an Intensive Care Unit: The Oxygen-ICU Randomized Clinical Trial. Jama. 2016;316(15):1583-9.

18. Barbateskovic M, Schjørring OL, Russo Krauss S, Jakobsen JC, Meyhoff CS, Dahl RM, et al. Higher versus lower fraction of inspired oxygen or targets of arterial oxygenation for adults admitted to the intensive care unit. Cochrane Database Syst Rev. 2019;2019(11).

19. Young P, Mackle D, Bellomo R, Bailey M, Beasley R, Deane A, et al. Conservative oxygen therapy for mechanically ventilated adults with sepsis: a post hoc analysis of data from the intensive care unit randomized trial comparing two approaches to oxygen therapy (ICU-ROX). Intensive Care Med. 2020;46(1):17-26.

20. Schjørring OL, Klitgaard TL, Perner A, Wetterslev J, Lange T, Siegemund M, et al. Lower or Higher Oxygenation Targets for Acute Hypoxemic Respiratory Failure. N Engl J Med. 2021;384(14):1301-11.

21. Barrot L, Asfar P, Mauny F, Winiszewski H, Montini F, Badie J, et al. Liberal or Conservative Oxygen Therapy for Acute Respiratory Distress Syndrome. N Engl J Med. 2020;382(11):999-1008.

22. Robba C, Battaglini D, Cinotti R, Asehnoune K, Stevens R, Taccone FS, et al. Individualized Thresholds of Hypoxemia and Hyperoxemia and their Effect on Outcome in Acute Brain Injured Patients: A Secondary Analysis of the ENIO Study. Neurocrit Care. 2023.

23. Sulzer CF, Chioléro R, Chassot PG, Mueller XM, Revelly JP. Adaptive support ventilation for fast tracheal extubation after cardiac surgery: a randomized controlled study. Anesthesiology. 2001;95(6):1339-45.

24. Petter AH, Chioléro RL, Cassina T, Chassot PG, Müller XM, Revelly JP. Automatic "respirator/weaning" with adaptive support ventilation: the effect on duration of endotracheal intubation and patient management. Anesth Analg. 2003;97(6):1743-50.

25. Jiang H, Yu SY, Wang LW. [Comparison of SmartCare and spontaneous breathing trials for weaning old patients with chronic obstructive pulmonary diseases]. Zhonghua Jie He He Hu Xi Za Zhi. 2006;29(8):545-8.

26. Rose L, Presneill JJ, Johnston L, Cade JF. A randomised, controlled trial of conventional versus automated weaning from mechanical ventilation using SmartCare/PS. Intensive Care Med. 2008;34(10):1788-95.

27. Xirouchaki N, Kondili E, Vaporidi K, Xirouchakis G, Klimathianaki M, Gavriilidis G, et al. Proportional assist ventilation with load-adjustable gain factors in critically ill patients: comparison with pressure support. Intensive Care Med. 2008;34(11):2026-34.

28. Dongelmans DA, Veelo DP, Paulus F, de Mol BA, Korevaar JC, Kudoga A, et al. Weaning automation with adaptive support ventilation: a randomized controlled trial in cardiothoracic surgery patients. Anesth Analg. 2009;108(2):565-71.

29. Stahl C, Dahmen G, Ziegler A, Muhl E. Comparison of automated protocol-based versus non-protocol-based physician-directed weaning from mechanical ventilation. Intensivmedizin und Notfallmedizin. 2009;46(6):441-6.

30. Ma YJ, Yang XJ, Cao XY, Ma XG. [Comparison of computer-driven weaning and physician-directed weaning from mechanical ventilation: a randomized prospective study]. Zhonghua Jie He He Hu Xi Za Zhi. 2010;33(3):174-8.

31. Coisel Y, Chanques G, Jung B, Constantin JM, Capdevila X, Matecki S, et al. Neurally adjusted ventilatory assist in critically ill postoperative patients: a crossover randomized study. Anesthesiology. 2010;113(4):925-35.

32. Kirakli C, Ozdemir I, Ucar ZZ, Cimen P, Kepil S, Ozkan SA. Adaptive support ventilation for faster weaning in COPD: a randomised controlled trial. Eur Respir J. 2011;38(4):774-80.

33. Arnal JM, Wysocki M, Novotni D, Demory D, Lopez R, Donati S, et al. Safety and efficacy of a fully closed-loop control ventilation (IntelliVent-ASV®) in sedated ICU patients with acute respiratory failure: a prospective randomized crossover study. Intensive Care Med. 2012;38(5):781-7.

34. Schädler D, Engel C, Elke G, Pulletz S, Haake N, Frerichs I, et al. Automatic control of pressure support for ventilator weaning in surgical intensive care patients. Am J Respir Crit Care Med. 2012;185(6):637-44.

35. Agarwal R, Srinivasan A, Aggarwal AN, Gupta D. Adaptive support ventilation for complete ventilatory support in acute respiratory distress syndrome: a pilot, randomized controlled trial. Respirology. 2013;18(7):1108-15.

36. Burns KE, Meade MO, Lessard MR, Hand L, Zhou Q, Keenan SP, Lellouche F. Wean earlier and automatically with new technology (the WEAN study). A multicenter, pilot randomized controlled trial. Am J Respir Crit Care Med. 2013;187(11):1203-11.

37. Clavieras N, Wysocki M, Coisel Y, Galia F, Conseil M, Chanques G, et al. Prospective randomized crossover study of a new closed-loop control system versus pressure support during weaning from mechanical ventilation. Anesthesiology. 2013;119(3):631-41.

38. Lellouche F, Bouchard PA, Simard S, L'Her E, Wysocki M. Evaluation of fully automated ventilation: a randomized controlled study in post-cardiac surgery patients. Intensive Care Med. 2013;39(3):463-71.

39. Liu L, Xu XT, Yang Y, Huang YZ, Liu SQ, Qiu HB. Computer-driven automated weaning reduces weaning duration in difficult-to-wean patients. Chin Med J (Engl). 2013;126(10):1814-8.

40. Celli P, Privato E, Ianni S, Babetto C, D'Arena C, Guglielmo N, et al. Adaptive support ventilation versus synchronized intermittent mandatory ventilation with pressure support in weaning patients after orthotopic liver transplantation. Transplant Proc. 2014;46(7):2272-8.

41. Elganady A, Beshey B, Abdelaziz A. Proportional assist ventilation versus pressure support ventilation in the weaning of patients with acute exacerbation of chronic obstructive pulmonary disease. Egyptian Journal of Chest Diseases and Tuberculosis. 2014;63.

42. Mohamed K, El Maraghi S. Role of Adaptive Support Ventilation in Weaning of COPD Patients. Egyptian Journal of Chest Diseases and Tuberculosis. 2014;63.

43. Kirakli C, Naz I, Ediboglu O, Tatar D, Budak A, Tellioglu E. A randomized controlled trial comparing the ventilation duration between adaptive support ventilation and pressure assist/control ventilation in medical patients in the ICU. Chest. 2015;147(6):1503-9.

44. Taniguchi C, Victor ES, Pieri T, Henn R, Santana C, Giovanetti E, et al. Smart Care™ versus respiratory physiotherapy-driven manual weaning for critically ill adult patients: a randomized controlled trial. Crit Care. 2015;19(1):246.

45. Teixeira SN, Osaku EF, Costa CR, Toccolini BF, Costa NL, Cândia MF, et al. Comparison of Proportional Assist Ventilation Plus, T-Tube Ventilation, and Pressure Support Ventilation as Spontaneous Breathing Trials for Extubation: A Randomized Study. Respir Care. 2015;60(11):1527-35.

46. Zhu F, Gomersall CD, Ng SK, Underwood MJ, Lee A. A randomized controlled trial of adaptive support ventilation mode to wean patients after fast-track cardiac valvular surgery. Anesthesiology. 2015;122(4):832-40.

47. Bialais E, Wittebole X, Vignaux L, Roeseler J, Wysocki M, Meyer J, et al. Closed-loop ventilation mode (IntelliVent®-ASV) in intensive care unit: a randomized trial. Minerva Anestesiol. 2016;82(6):657-68.

48. Bosma KJ, Read BA, Bahrgard Nikoo MJ, Jones PM, Priestap FA, Lewis JF. A Pilot Randomized Trial Comparing Weaning From Mechanical Ventilation on Pressure Support Versus Proportional Assist Ventilation. Crit Care Med. 2016;44(6):1098-108.

49. Demoule A, Clavel M, Rolland-Debord C, Perbet S, Terzi N, Kouatchet A, et al. Neurally adjusted ventilatory assist as an alternative to pressure support ventilation in adults: a French multicentre randomized trial. Intensive Care Med. 2016;42(11):1723-32.

50. Yazdannik A, Zarei H, Massoumi G. Comparing the effects of adaptive support ventilation and synchronized intermittent mandatory ventilation on intubation duration and hospital stay after coronary artery bypass graft surgery. Iran J Nurs Midwifery Res. 2016;21(2):207-12.

51. Fot EV, Izotova NN, Yudina AS, Smetkin AA, Kuzkov VV, Kirov MY. Automated Weaning from Mechanical Ventilation after Off-Pump Coronary Artery Bypass Grafting. Front Med (Lausanne). 2017;4:31.

52. Moradian ST, Saeid Y, Ebadi A, Hemmat A, Ghiasi MS. Adaptive Support Ventilation Reduces the Incidence of Atelectasis in Patients Undergoing Coronary Artery Bypass Grafting: A Randomized Clinical Trial. Anesth Pain Med. 2017;7(3):e44619.

53. Arnal JM, Garnero A, Novotni D, Corno G, Donati SY, Demory D, et al. Closed loop ventilation mode in Intensive Care Unit: a randomized controlled clinical trial comparing the numbers of manual ventilator setting changes. Minerva Anestesiol. 2018;84(1):58-67.

54. Botha J, Green C, Carney I, Haji K, Gupta S, Tiruvoipati R. Proportional assist ventilation versus pressure support ventilation in weaning ventilation: a pilot randomised controlled trial. Crit Care Resusc. 2018;20(1):33-40.

55. Grieco DL, Bitondo MM, Aguirre-Bermeo H, Italiano S, Idone FA, Moccaldo A, et al. Patient-ventilator interaction with conventional and automated management of pressure support during difficult weaning from mechanical ventilation. J Crit Care. 2018;48:203-10.

56. Delgado M, Subirá C, Hermosa C, Gordo F, Riera J, Fernández R. Proportional assist ventilation feasibility in the early stage of respiratory failure: a prospective randomized multicenter trial. Minerva Anestesiol. 2019;85(8):862-70.

57. De Bie AJR, Neto AS, van Meenen DM, Bouwman AR, Roos AN, Lameijer JR, et al. Fully automated postoperative ventilation in cardiac surgery patients: a randomised clinical trial. Br J Anaesth. 2020;125(5):739-49.

58. Chelly J, Mazerand S, Jochmans S, Weyer CM, Pourcine F, Ellrodt O, et al. Automated vs. conventional ventilation in the ICU: a randomized controlled crossover trial comparing blood oxygen saturation during daily nursing procedures (I-NURSING). Crit Care. 2020;24(1):453.

59. Diniz-Silva F, Moriya HT, Alencar AM, Amato MBP, Carvalho CRR, Ferreira JC. Neurally adjusted ventilatory assist vs. pressure support to deliver protective mechanical ventilation in patients with acute respiratory distress syndrome: a randomized crossover trial. Ann Intensive Care. 2020;10(1):18.

60. Eremenko A, Komnov R. Smart Mode of Mechanical Lung Ventilation During Early Activation of Cardiosurgical Patients. General Reanimatology. 2020;16:4-15.

61. Hadfield DJ, Rose L, Reid F, Cornelius V, Hart N, Finney C, et al. Neurally adjusted ventilatory assist versus pressure support ventilation: a randomized controlled feasibility trial performed in patients at risk of prolonged mechanical ventilation. Crit Care. 2020;24(1):220.

62. Kacmarek RM, Villar J, Parrilla D, Alba F, Solano R, Liu S, et al. Neurally adjusted ventilatory assist in acute respiratory failure: a randomized controlled trial. Intensive Care Med. 2020;46(12):2327-37.

63. Liu L, Xu X, Sun Q, Yu Y, Xia F, Xie J, et al. Neurally Adjusted Ventilatory Assist versus Pressure Support Ventilation in Difficult Weaning: A Randomized Trial. Anesthesiology. 2020;132(6):1482-93.

64. Cammarota G, Verdina F, De Vita N, Boniolo E, Tarquini R, Messina A, et al. Effects of Varying Levels of Inspiratory Assistance with Pressure Support Ventilation and Neurally Adjusted Ventilatory Assist on Driving Pressure in Patients Recovering from Hypoxemic Respiratory Failure. J Clin Monit Comput. 2022;36(2):419-27.

65. Baedorf Kassis EN, Bastos AB, Schaefer MS, Capers K, Hoenig B, Banner-Goodspeed V, Talmor D. Adaptive Support Ventilation and Lung-Protective Ventilation in ARDS. Respir Care. 2022;67(12):1542-50.

66. Sehgal IS, Gandra RR, Dhooria S, Aggarwal AN, Prasad KT, Muthu V, et al. A randomised trial of adaptive support ventilation in patients with neuroparalytic snake envenomation. Br J Anaesth. 2022;128(3):e232-e4.

67. Zhang J, Yang Z, Chen K, Zhang X, Zhao T, Zhang X. Efficacy of adaptive ventilation support combined with lung recruitment maneuvering for acute respiratory distress syndrome. Am J Transl Res. 2022;14(3):2109-16.

68. Claure N, Bancalari E, D'Ugard C, Nelin L, Stein M, Ramanathan R, et al. Multicenter crossover study of automated control of inspired oxygen in ventilated preterm infants. Pediatrics. 2011;127(1):e76-83.

69. Jouvet PA, Payen V, Gauvin F, Emeriaud G, Lacroix J. Weaning children from mechanical ventilation with a computer-driven protocol: a pilot trial. Intensive Care Med. 2013;39(5):919-25.

70. Lal M, Tin W, Sinha S. Automated control of inspired oxygen in ventilated preterm infants: crossover physiological study. Acta Paediatr. 2015;104(11):1084-9.

71. van Kaam AH, Hummler HD, Wilinska M, Swietlinski J, Lal MK, te Pas AB, et al. Automated versus Manual Oxygen Control with Different Saturation Targets and Modes of Respiratory Support in Preterm Infants. J Pediatr. 2015;167(3):545-50.e1-2.

72. Kallio M, Peltoniemi O, Anttila E, Pokka T, Kontiokari T. Neurally adjusted ventilatory assist (NAVA) in pediatric intensive care--a randomized controlled trial. Pediatr Pulmonol. 2015;50(1):55-62.

73. Soydan E, Ceylan G, Topal S, Hepduman P, Atakul G, Colak M, et al. Automated closed-loop FiO(2) titration increases the percentage of time spent in optimal zones of oxygen saturation in pediatric patients-A randomized crossover clinical trial. Front Med (Lausanne). 2022;9:969218.
